# Mortality Trends Involving Cardiac Arrest and Fluid, Electrolyte, and Acid–Base Disorders in U.S. Adults, 2004–2024

**DOI:** 10.3390/jcm15166258

**Published:** 2026-08-13

**Authors:** Hassaan Abid, Govinda Lohano, Bhawan Kumar, Kinza Irshad, Gaaitri Lohano, Fizzah Ikram Ul Haq, Mukesh Meghwar, Moiz Ul Haq Hashmi

**Affiliations:** 1 Department of Internal Medicine, Indiana University School of Medicine, Indianapolis, IN 46202, USA; 2 Department of Internal Medicine, Liaquat University of Medical and Health Sciences, Jamshoro 76090, Pakistan; govindalohano7@gmail.com (G.L.); gaitrilohano@gmail.com (G.L.); 3 Department of Internal Medicine, Dow University of Health Sciences, Karachi 75600, Pakistan; bhawankumar0345@gmail.com; 4 Department of Internal Medicine, Ziauddin Medical College, Karachi 75600, Pakistan; ikinza145@gmail.com; 5 Department of Internal Medicine, Allama Iqbal Medical College, Lahore 54550, Pakistan; fizzahikramulhaq@gmail.com; 6 Department of Internal Medicine, Gambat Institute of Medical Sciences, Khairpur 66070, Pakistan; mukeshvalasai8@gmail.com; 7 Department of Internal Medicine, Nishtar Medical University, Multan 66000, Pakistan; moizhashmi1424@gmail.com

**Keywords:** cardiac arrest, electrolyte disorders, acid–base disorders, mortality trends, CDC WONDER

## Abstract

**Background/Objectives:** Cardiac arrest and fluid, electrolyte, and acid–base disorders frequently coexist in critically ill patients and are associated with poor clinical outcomes. However, long-term population-level trends in mortality involving both conditions remain poorly characterized. This study evaluated temporal trends and demographic disparities in mortality involving cardiac arrest and fluid, electrolyte, and acid–base disorders among U.S. adults from 2004 to 2024. **Methods:** We conducted a retrospective population-based study using the Centers for Disease Control and Prevention Wide-Ranging Online Data for Epidemiologic Research (CDC WONDER) Multiple Cause of Death database. Adults aged ≥25 years with cardiac arrest (ICD-10: I46) and fluid, electrolyte, and acid–base disorders (ICD-10: E87) listed anywhere on the same death certificate between 2004 and 2024 were included. Age-adjusted mortality rates (AAMRs) were calculated using the 2000 U.S. standard population. Joinpoint regression was used to estimate annual percent change (APC) and average annual percent change (AAPC), with analyses stratified by demographic and geographic characteristics. **Results:** A total of 152,937 deaths involved both cardiac arrest and fluid, electrolyte, and acid–base disorders during the study period. The AAMR nearly doubled, increasing from 2.06 (95% CI, 2.00–2.13) in 2004 to 4.08 (95% CI, 4.00–4.16) in 2024, corresponding to an overall AAPC of 3.42% (95% CI, 2.58–4.26; *p* < 0.001). Mortality remained consistently higher among males, adults aged ≥65 years, and non-Hispanic Black individuals, with significant increases observed across all demographic groups. The West demonstrated the highest regional mortality burden (AAPC, 3.75%; *p* < 0.001). Urbanization analyses (2004–2020) showed higher mortality in non-metropolitan than metropolitan areas (AAPCs, 5.36% vs. 4.13%; both *p* < 0.001). Mortality increased through 2021 before declining modestly during 2021–2024. **Conclusions:** Mortality involving both cardiac arrest and fluid, electrolyte, and acid–base disorders increased substantially in the United States over the past two decades, with persistent disparities in age, sex, race/ethnicity, and geography. These findings identify populations at increased risk and support targeted public health strategies, equitable healthcare access, and improved recognition and management of metabolic disturbances in patients at risk for cardiac arrest.

## 1. Introduction

Cardiac arrest is a sudden and abrupt loss of heart function due to failure of the heart’s electrical system, leading to the absence of normal breathing and signs of circulation [1]. In the United States, it remains a major public health problem and a persistent cause of mortality despite advances in emergency response and cardiovascular care [1,2]. Although overall mortality has declined over time, the burden is not equally distributed, with studies using CDC WONDER data suggesting that men, racial and ethnic minorities, and rural populations continue to experience a higher risk [2,3].

Electrolyte and acid–base disorders represent another important clinical burden because they are common in hospitalized and medically ill patients and are associated with serious cardiovascular complications [4,5]. Disturbances in potassium, sodium, calcium, and acid–base balance can alter cardiac electrophysiology, impair myocardial function, and contribute to arrhythmia development and hemodynamic instability. These electrolyte disturbances modify transmembrane ionic currents and disrupt normal impulse generation and conduction, thereby promoting electrical instability and increasing susceptibility to clinically significant arrhythmias that may lead to cardiac arrest [6]. National studies have also reported that mortality related to electrolyte and acid–base imbalance in the United States may be increasing, highlighting the need for population-level assessment [7].

Laboratory and clinical studies further demonstrate that these metabolic derangements occur dynamically during cardiac arrest and resuscitation, reflecting a close physiological link between electrolyte imbalance and cardiovascular collapse [8]. This close overlap suggests that cardiac arrest and electrolyte and acid–base disorders should be examined in an integrated manner rather than as isolated conditions, while recognizing that their coexistence represents a clinically important association rather than evidence of a direct causal relationship.

Despite this biological link, there is limited evidence describing long-term temporal trends of cardiac arrest and electrolyte and acid–base disorder–related mortality in the United States. Population-level analysis can help identify changes over time and disparities across subgroups. Therefore, this study aims to evaluate epidemiological trends in cardiac arrest and electrolyte and acid–base disorders in the United States from 2004 to 2024 to inform prevention strategies, surveillance, and clinical planning.

## 2. Methods

### 2.1. Study Population and Setting

This retrospective population-based study utilized mortality data obtained from the Centers for Disease Control and Prevention’s (CDC) Wide-Ranging Online Data for Epidemiologic Research (CDC WONDER) Multiple Cause of Death (MCOD) database [9] to evaluate epidemiological trends in mortality associated with cardiac arrest and electrolyte and acid–base disorders in the United States. The analysis was restricted to the period from 2004 to 2024 because mortality estimates for earlier years contained unreliable or suppressed data within the CDC WONDER database, limiting the ability to generate stable and interpretable trend estimates. Restricting the analysis to 2004 onward ensured more complete and reliable mortality reporting throughout the study period. Cases were identified using the International Classification of Diseases, Tenth Revision (ICD-10) codes I46 (cardiac arrest) and E87 (disorders of fluid, electrolyte, and acid–base balance) [7,10]. Death certificates from adults aged ≥25 years were included in the analysis. The Multiple Cause of Death Public Use Records were examined to identify deaths in which both cardiac arrest (ICD-10: I46) and disorders of fluid, electrolyte, and acid–base balance (ICD-10: E87) were recorded anywhere on the same death certificate, regardless of whether they were listed as the underlying or contributing causes of death. This approach was selected to better characterize the overall mortality burden associated with these conditions, as analyses restricted solely to underlying causes of death may underestimate their contribution to population-level mortality. Institutional Review Board approval was not required because the CDC WONDER database contains publicly available and deidentified data. All study procedures were conducted in accordance with the Strengthening the Reporting of Observational Studies in Epidemiology (STROBE) guidelines [11].

### 2.2. Data Extraction

Extracted variables included year of death, age group, sex, race/ethnicity, U.S. state of residence, census region, urbanization status, and place of death. Age was categorized into 25–44 years, 45–64 years, 65–74 years, 75–84 years, and ≥85 years. Sex was classified as male or female according to death certificate records available in the CDC WONDER database. Race and ethnicity were categorized according to CDC WONDER classifications as non-Hispanic White, non-Hispanic Black or African American, non-Hispanic Asian or Pacific Islander, non-Hispanic American Indian or Alaska Native, and Hispanic or Latino. Geographic analyses were performed according to U.S. Census Bureau regional classifications, including the Northeast, Midwest, South, and West. Urbanization status was classified using the 2013 National Center for Health Statistics Urban–Rural Classification Scheme and grouped into metropolitan and non-metropolitan areas [12]. Urbanization analyses were restricted to 2004–2020 because county-level urbanization classifications were not available in the CDC WONDER database for subsequent years at the time of analysis. Place of death was categorized as medical facilities (outpatient, emergency department, inpatient, dead on arrival, or status unknown), home, hospice facility, and nursing or long-term care facility.

### 2.3. Statistical Analysis

Crude rate and age-adjusted mortality rates (AAMRs) per 100,000 population were calculated using CDC WONDER standard methodology. AAMRs were computed using the direct age-standardization method based on the 2000 U.S. standard population to account for differences in age distribution [13]. Temporal trends in mortality from 2004 to 2024 were evaluated using the Joinpoint Regression Program (Version 5.4.0, National Cancer Institute, Bethesda, MD, USA), which fits log-linear regression models to identify statistically significant changes in mortality trends over time [14]. The joinpoint regression model employed permutation tests with standard error adjustments as implemented in the National Cancer Institute software. Annual percent change (APC) was calculated for each identified trend segment, whereas average annual percent change (AAPC) was calculated for the overall study period. APC and AAPC estimates were reported with corresponding 95% confidence intervals (CIs). APC and AAPC values were considered statistically significant if the two-sided *p*-value was <0.05. Additionally, a supplementary analysis was performed by stratifying deaths according to individual ICD-10 E87 sub-codes to evaluate the contribution of specific electrolyte and acid–base disorders to the overall mortality trends. ICD-10 sub-codes E87.3 (alkalosis), E87.4 (mixed disorder of acid–base balance), and E87.7 (fluid overload) were excluded from this analysis because of unreliable mortality data, which precluded robust trend estimation.

## 3. Results

### 3.1. Overall

**Figure 1:**
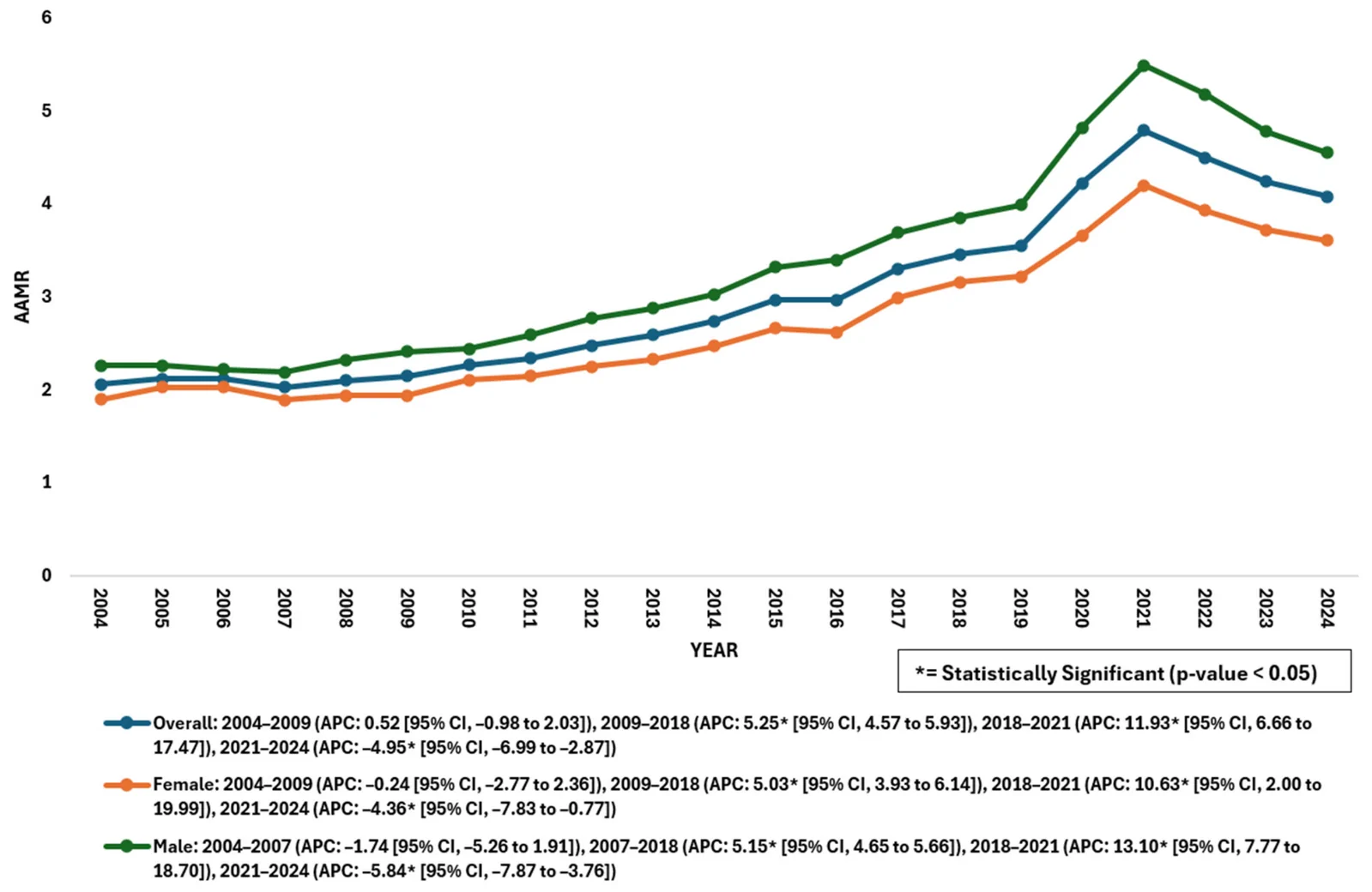
Overall and sex-stratified age-adjusted mortality rates (AAMRs) for mortality involving both cardiac arrest and electrolyte and acid–base disorders among U.S. adults aged ≥ 25 years, 2004–2024.

A total of 152,937 deaths were recorded during 2004–2024, including 77,450 (50.6%) females and 75,487 (49.4%) males. Overall, AAMR increased from 2.06 (95% CI: 2.00 to 2.13) in 2004 to 4.08 (95% CI: 4.00 to 4.16) in 2024. Joinpoint regression identified a non-significant increase from 2004 to 2009 (APC: 0.52; 95% CI: −0.98 to 2.03; *p* = 0.461), followed by a significant rise from 2009 to 2018 (APC: 5.25; 95% CI: 4.57 to 5.93; *p* < 0.000001). A sharp acceleration was observed from 2018 to 2021 (APC: 11.93; 95% CI: 6.66 to 17.47; *p* = 0.0004), followed by a significant decline from 2021 to 2024 (APC: −4.95; 95% CI: −6.99 to −2.87; *p* = 0.000387). The overall AAPC for 2004–2024 was 3.42 (95% CI: 2.58 to 4.26; *p* < 0.000001) (Figure 1, Supplementary Tables S1 and S8).

A supplementary analysis of individual ICD-10 E87 sub-codes was performed to evaluate the contribution of specific electrolyte and acid–base disorders to the observed mortality trends. Detailed results are presented in Supplementary Table S9 and Figure S4.

### 3.2. Stratified by Gender

In females, AAMR increased from 1.90 (95% CI: 1.82 to 1.98) in 2004 to 3.61 (95% CI: 3.52 to 3.71) in 2024, with an AAPC of 3.04 (95% CI: 1.64 to 4.46; *p* = 0.000018). Trends showed stability from 2004 to 2009 (APC: −0.24; 95% CI: −2.77 to 2.36; *p* = 0.840), followed by a significant increase from 2009 to 2018 (APC: 5.03; 95% CI: 3.93 to 6.14; *p* < 0.000001). A rapid rise occurred from 2018 to 2021 (APC: 10.63; 95% CI: 2.00 to 19.99; *p* = 0.019), followed by a significant decline from 2021 to 2024 (APC: −4.36; 95% CI: −7.83 to −0.77; *p* = 0.022).

In males, AAMR increased from 2.26 (95% CI: 2.15 to 2.37) in 2004 to 4.55 (95% CI: 4.43 to 4.67) in 2024, with an AAPC of 3.50 (95% CI: 2.60 to 4.42; *p* < 0.000001). A non-significant decline was observed from 2004 to 2007 (APC: −1.74; 95% CI: −5.26 to 1.91; *p* = 0.308), followed by a significant increase from 2007 to 2018 (APC: 5.15; 95% CI: 4.65 to 5.66; *p* < 0.000001). A sharp rise occurred from 2018 to 2021 (APC: 13.10; 95% CI: 7.77 to 18.70; *p* = 0.000205), followed by a significant decline from 2021 to 2024 (APC: −5.84; 95% CI: −7.87 to −3.76; *p* = 0.000109) (Figure 1, Supplementary Tables S1 and S8).

### 3.3. Stratified by Age

Among age groups, the highest mean CMR was observed in ≥85 years (28.19), followed by 75–84 years (11.81), 65–74 years (5.74), 45–64 years (2.25) and 25–44 years (0.42).

Among adults aged ≥85 years, the highest CMRs were observed throughout the study period, increasing from 24.11 (95% CI: 22.68 to 25.54) in 2004 to 33.30 (95% CI: 31.89 to 34.71) in 2024. The overall trend showed a modest but significant increase (AAPC: 1.75%; 95% CI: 0.74 to 2.77; *p* = 0.000685). Mortality remained relatively stable from 2004 to 2014 (APC: 0.45%; 95% CI: −0.57 to 1.48; *p* = 0.361), increased significantly during 2014–2021 (APC: 5.85%; 95% CI: 3.96 to 7.77; *p* = 0.000012), and subsequently showed a non-significant decline during 2021–2024 (APC: −3.14%; 95% CI: −7.86 to 1.83; *p* = 0.192).

Among individuals aged 75–84 years, the CMR increased from 8.59 (95% CI: 8.09 to 9.10) in 2004 to 15.40 (95% CI: 14.85 to 15.96) in 2024, with a significant overall increase (AAPC: 2.73%; 95% CI: 1.18 to 4.30; *p* = 0.000497). Mortality remained stable between 2004 and 2007 (APC: −0.96%; 95% CI: −6.88 to 5.34; *p* = 0.735), followed by significant increases during 2007–2018 (APC: 3.67%; 95% CI: 2.80 to 4.54; *p* = 0.000002) and 2018–2021 (APC: 11.67%; 95% CI: 2.65 to 21.48; *p* = 0.015), before declining significantly during 2021–2024 (APC: −5.18%; 95% CI: −8.63 to −1.61; *p* = 0.009).

Among individuals aged 65–74 years, the CMR increased from 3.67 (95% CI: 3.39 to 3.94) in 2004 to 8.00 (95% CI: 7.71 to 8.30) in 2024, with a significant overall increase (AAPC: 4.00%; 95% CI: 2.20 to 5.83; *p* = 0.000011). After a non-significant change during 2004–2007 (APC: −1.91%; 95% CI: −9.75 to 6.61; *p* = 0.617), mortality increased significantly during 2007–2018 (APC: 6.15%; 95% CI: 5.09 to 7.21; *p* < 0.000001) and 2018–2021 (APC: 11.22%; 95% CI: 1.95 to 21.34; *p* = 0.021), followed by a modest but significant decline during 2021–2024 (APC: −4.33%; 95% CI: −8.11 to −0.40; *p* = 0.034).

Among individuals aged 45–64 years, the CMR increased from 1.18 (95% CI: 1.10 to 1.26) in 2004 to 3.43 (95% CI: 3.30 to 3.55) in 2024, with a significant overall increase (AAPC: 5.42%; 95% CI: 4.06 to 6.80; *p* < 0.000001). Mortality increased significantly during 2004–2011 (APC: 3.22%; 95% CI: 0.59 to 5.91; *p* = 0.020) and more rapidly during 2011–2021 (APC: 9.79%; 95% CI: 8.28 to 11.32; *p* < 0.000001), before showing a non-significant decline between 2021 and 2024 (APC: −3.26%; 95% CI: −8.67 to 2.47; *p* = 0.236).

**Figure 2:**
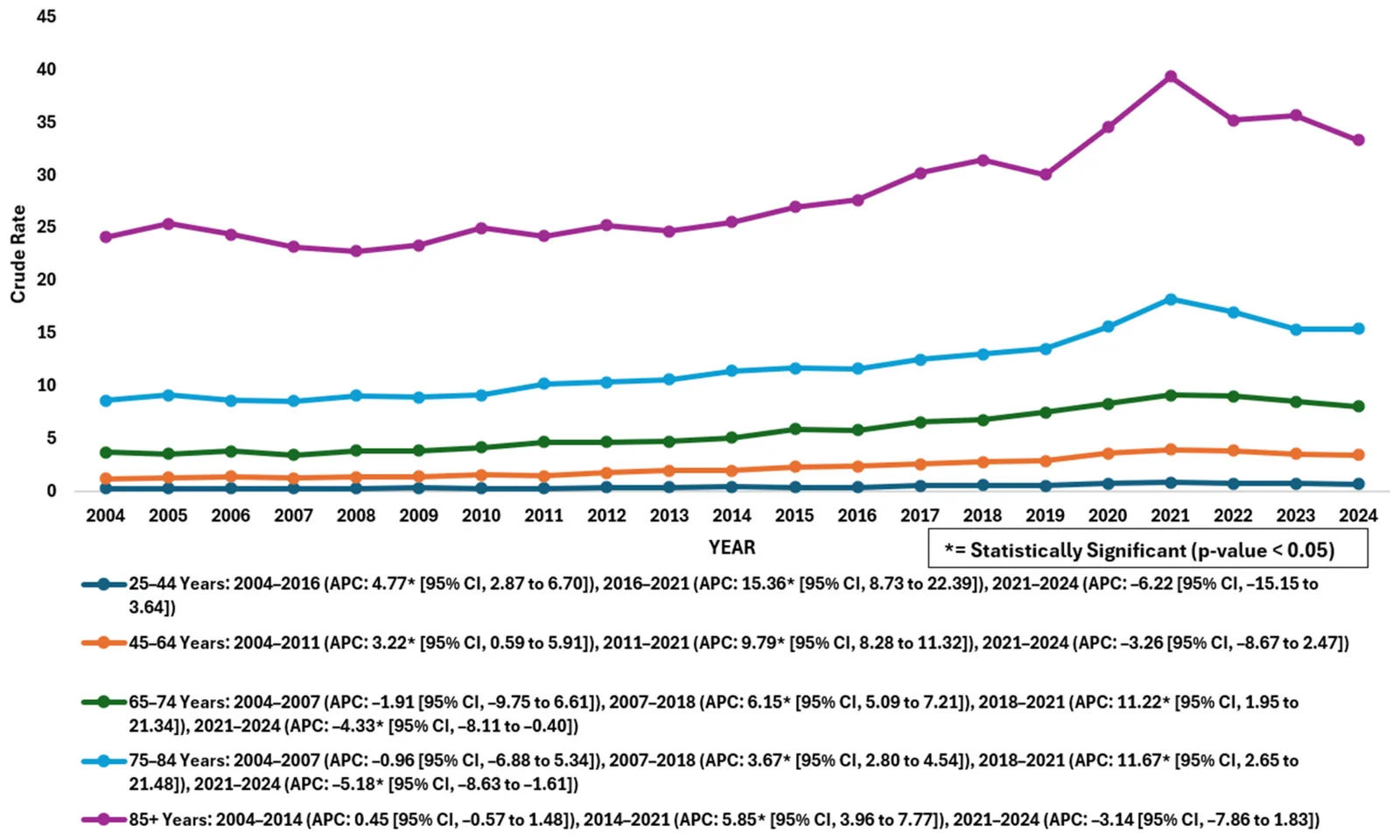
Age-stratified crude mortality rates (CMRs) for mortality involving both cardiac arrest and electrolyte and acid–base disorders among U.S. adults aged ≥25 years, 2004–2024.

Among adults aged 25–44 years, the CMR increased from 0.24 (95% CI: 0.21 to 0.28) in 2004 to 0.64 (95% CI: 0.59 to 0.69) in 2024, with a significant overall increase (AAPC: 5.55%; 95% CI: 3.30 to 7.85; *p* < 0.000001). Mortality increased significantly from 2004 to 2016 (APC: 4.77%; 95% CI: 2.87 to 6.70; *p* = 0.000099) and accelerated during 2016–2021 (APC: 15.36%; 95% CI: 8.73 to 22.39; *p* = 0.000166), followed by a non-significant decline from 2021 to 2024 (APC: −6.22%; 95% CI: −15.15 to 3.64; *p* = 0.189; Figure 2, Supplementary Tables S2 and S8).

### 3.4. Stratified by Race/Ethnicity

Across groups, the highest mean AAMR was observed in Black populations (5.27), followed by American Indian or Alaska Native (5.02), Hispanic/Latino (3.34), White (2.67), and Asian or Pacific Islander (2.58).

Among Black individuals, AAMR increased from 3.84 (95% CI: 3.54 to 4.14) in 2004 to 7.46 (95% CI: 7.14 to 7.80) in 2024, with an AAPC of 3.05 (95% CI: 1.78 to 4.33; *p* = 0.000002). A non-significant decline was observed from 2004 to 2008 (APC: −3.44; 95% CI: −6.85 to 0.09; *p* = 0.055), followed by a significant increase from 2008 to 2018 (APC: 5.11; 95% CI: 4.22 to 6.00; *p* < 0.000001). A sharp increase occurred from 2018 to 2021 (APC: 13.12; 95% CI: 5.58 to 21.20; *p* = 0.002597), followed by a significant decline from 2021 to 2024 (APC: −4.16; 95% CI: −7.11 to −1.12; *p* = 0.012697).

Among American Indian or Alaska Native individuals, AAMR increased from 3.22 (95% CI: 2.12 to 4.68) in 2004 to 6.64 (95% CI: 5.47 to 8.02) in 2024, with an AAPC of 4.67 (95% CI: 2.37 to 7.01; *p* = 0.000054). A significant increase was observed from 2004 to 2022 (APC: 6.10; 95% CI: 4.98 to 7.23; *p* < 0.000001), followed by a non-significant decline from 2022 to 2024 (APC: −7.38; 95% CI: −25.65 to 15.37; *p* = 0.470).

Among Hispanic individuals, AAMR increased from 2.39 (95% CI: 2.10 to 2.69) in 2004 to 4.61 (95% CI: 4.37 to 4.86) in 2024, with an AAPC of 2.94 (95% CI: 0.86 to 5.06; *p* = 0.00536). A non-significant decline was observed from 2004 to 2008 (APC: −2.62; 95% CI: −8.94 to 4.13; *p* = 0.398), followed by a significant increase from 2008 to 2018 (APC: 4.37; 95% CI: 2.89 to 5.87; *p* = 0.000056). A sharp increase occurred from 2018 to 2021 (APC: 15.98; 95% CI: 4.27 to 29.01; *p* = 0.011), followed by a significant decline from 2021 to 2024 (APC: −6.04; 95% CI: −10.38 to −1.49; *p* = 0.015).

Among White individuals, AAMR increased from 1.84 (95% CI: 1.77 to 1.91) in 2004 to 3.51 (95% CI: 3.42 to 3.60) in 2024, with an AAPC of 3.32 (95% CI: 2.31 to 4.33; *p* < 0.000001). A non-significant increase was observed from 2004 to 2009 (APC: 0.79; 95% CI: −0.95 to 2.56; *p* = 0.338), followed by a significant increase from 2009 to 2018 (APC: 5.29; 95% CI: 4.46 to 6.13; *p* < 0.000001). A sharp increase occurred from 2018 to 2021 (APC: 10.25; 95% CI: 4.13 to 16.73; *p* = 0.003449), followed by a significant decline from 2021 to 2024 (APC: −4.67; 95% CI: −7.41 to −1.85; *p* = 0.004434).

**Figure 3:**
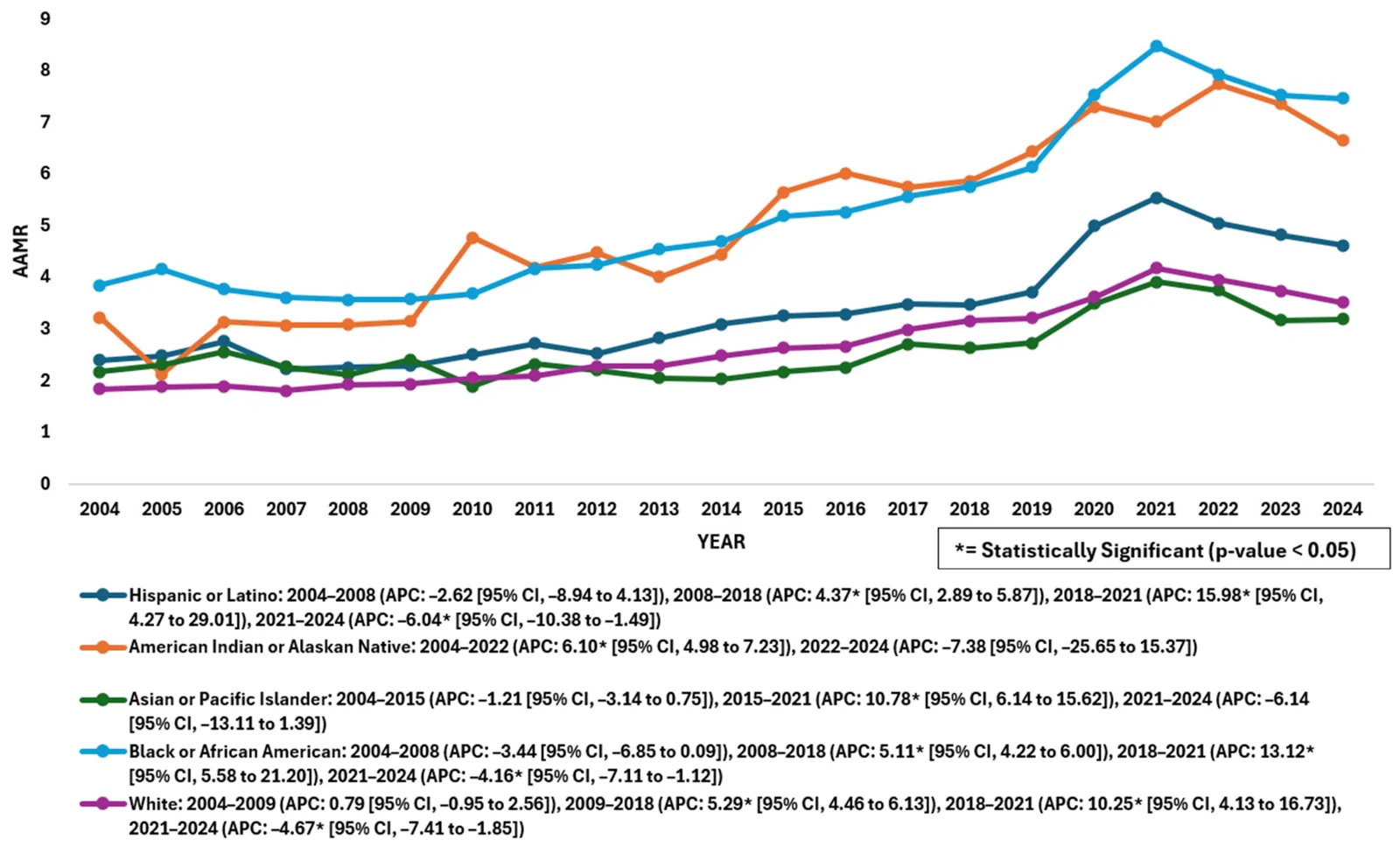
Race/ethnicity-stratified age-adjusted mortality rates (AAMRs) for mortality involving both cardiac arrest and electrolyte and acid–base disorders among U.S. adults aged ≥25 years, 2004–2024.

Among Asian or Pacific Islander individuals, AAMR increased from 2.17 (95% CI: 1.77 to 2.58) in 2004 to 3.19 (95% CI: 2.92 to 3.48) in 2024. The overall trend was not statistically significant (AAPC: 1.46; 95% CI: −0.40 to 3.35; *p* = 0.126). A non-significant decline was observed from 2004 to 2015 (APC: −1.21; 95% CI: −3.14 to 0.75; *p* = 0.204), followed by a significant increase from 2015 to 2021 (APC: 10.78; 95% CI: 6.14 to 15.62; *p* = 0.000181). A non-significant decline occurred from 2021 to 2024 (APC: −6.14; 95% CI: −13.11 to 1.39; *p* = 0.099) (Figure 3, Supplementary Tables S3 and S8).

### 3.5. Stratified by Census Region

Across regions, the highest mean AAMR was observed in the West (3.54), followed by the Northeast (3.43), South (2.93), and Midwest (2.19).

In the West, AAMR increased from 2.52 (95% CI: 2.37 to 2.68) in 2004 to 4.83 (95% CI: 4.66 to 5.00) in 2024, with an AAPC of 3.75 (95% CI: 2.72 to 4.79; *p* < 0.000001). A significant increase was observed from 2004 to 2014 (APC: 1.59; 95% CI: 0.40 to 2.80; *p* = 0.012834), followed by a marked increase from 2014 to 2021 (APC: 11.04; 95% CI: 9.05 to 13.06; *p* < 0.000001). A significant decline occurred from 2021 to 2024 (APC: −5.00; 95% CI: −9.20 to −0.62; *p* = 0.028902).

In the Northeast, AAMR increased from 2.41 (95% CI: 2.26 to 2.57) in 2004 to 4.83 (95% CI: 4.64 to 5.03) in 2024, with an AAPC of 3.35 (95% CI: 1.99 to 4.73; *p* = 0.000001). A non-significant increase was observed from 2004 to 2009 (APC: 0.86; 95% CI: −1.66 to 3.45; *p* = 0.468), followed by a significant increase from 2009 to 2018 (APC: 4.20; 95% CI: 3.11 to 5.29; *p* = 0.000005). A sharp increase occurred from 2018 to 2021 (APC: 11.99; 95% CI: 3.57 to 21.10; *p* = 0.009042), followed by a non-significant decline from 2021 to 2024 (APC: −3.07; 95% CI: −6.47 to 0.45; *p* = 0.080).

In the South, AAMR increased from 1.93 (95% CI: 1.83 to 2.04) in 2004 to 3.85 (95% CI: 3.73 to 3.98) in 2024, with an AAPC of 3.63 (95% CI: 2.09 to 5.19; *p* = 0.000003). A significant increase was observed from 2004 to 2018 (APC: 3.94; 95% CI: 3.30 to 4.57; *p* < 0.000001), followed by a sharp increase from 2018 to 2021 (APC: 12.89; 95% CI: 2.59 to 24.22; *p* = 0.016919). A significant decline occurred from 2021 to 2024 (APC: −6.19; 95% CI: −10.39 to −1.79; *p* = 0.00999).

**Figure 4:**
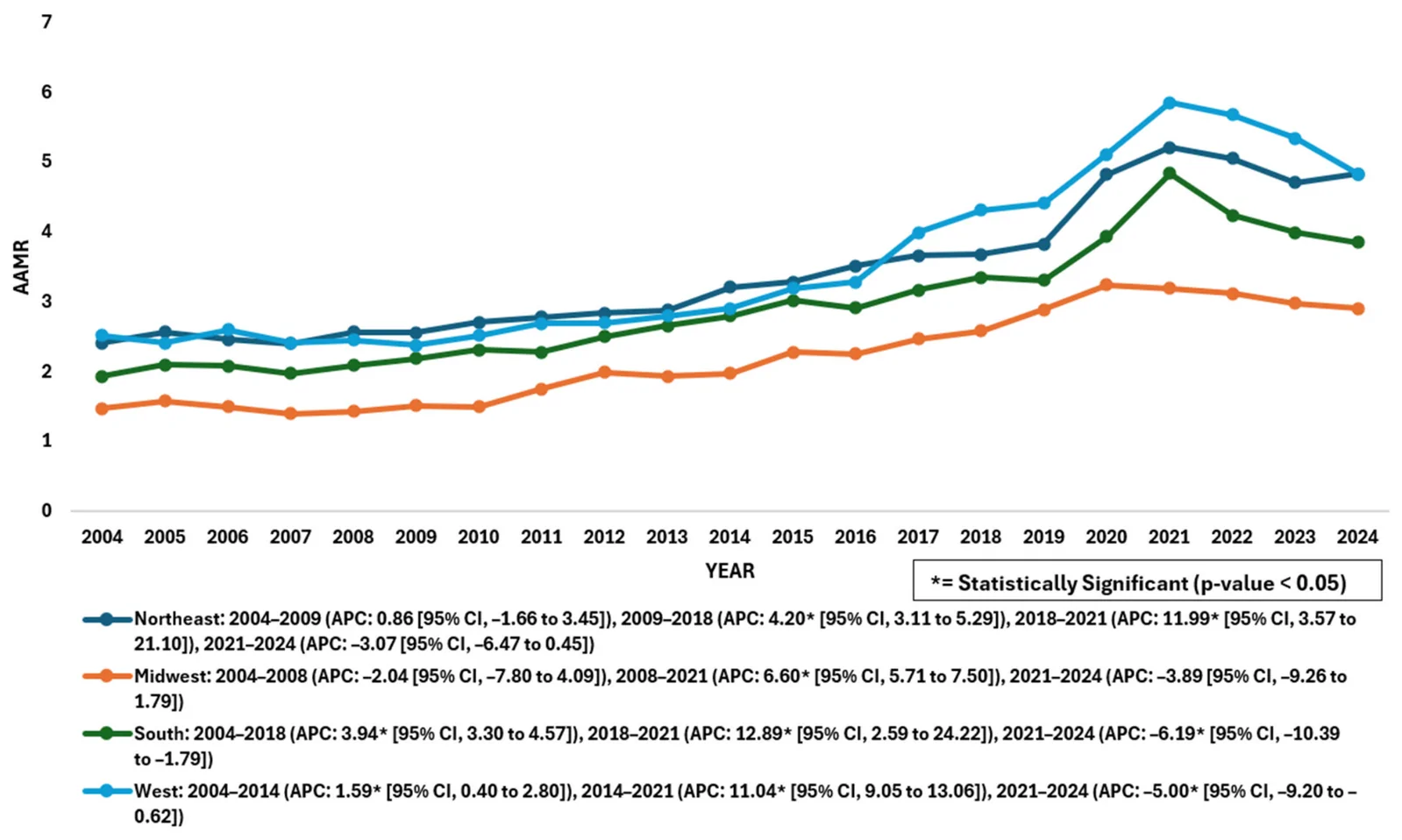
Census region-stratified age-adjusted mortality rates (AAMRs) for mortality involving both cardiac arrest and electrolyte and acid–base disorders among U.S. adults aged ≥25 years, 2004–2024.

In the Midwest, AAMR increased from 1.47 (95% CI: 1.36 to 1.59) in 2004 to 2.90 (95% CI: 2.76 to 3.04) in 2024, with an AAPC of 3.20 (95% CI: 1.73 to 4.69; *p* = 0.000018). A non-significant decline was observed from 2004 t o 2008 (APC: −2.04; 95% CI: −7.80 to 4.09; *p* = 0.476), followed by a significant increase from 2008 to 2021 (APC: 6.60; 95% CI: 5.71 to 7.50; *p* < 0.000001). A non-significant decline occurred from 2021 to 2024 (APC: −3.89; 95% CI: −9.26 to 1.79; *p* = 0.160) (Figure 4, Supplementary Tables S4 and S8).

### 3.6. Stratified by Urbanization

**Figure 5:**
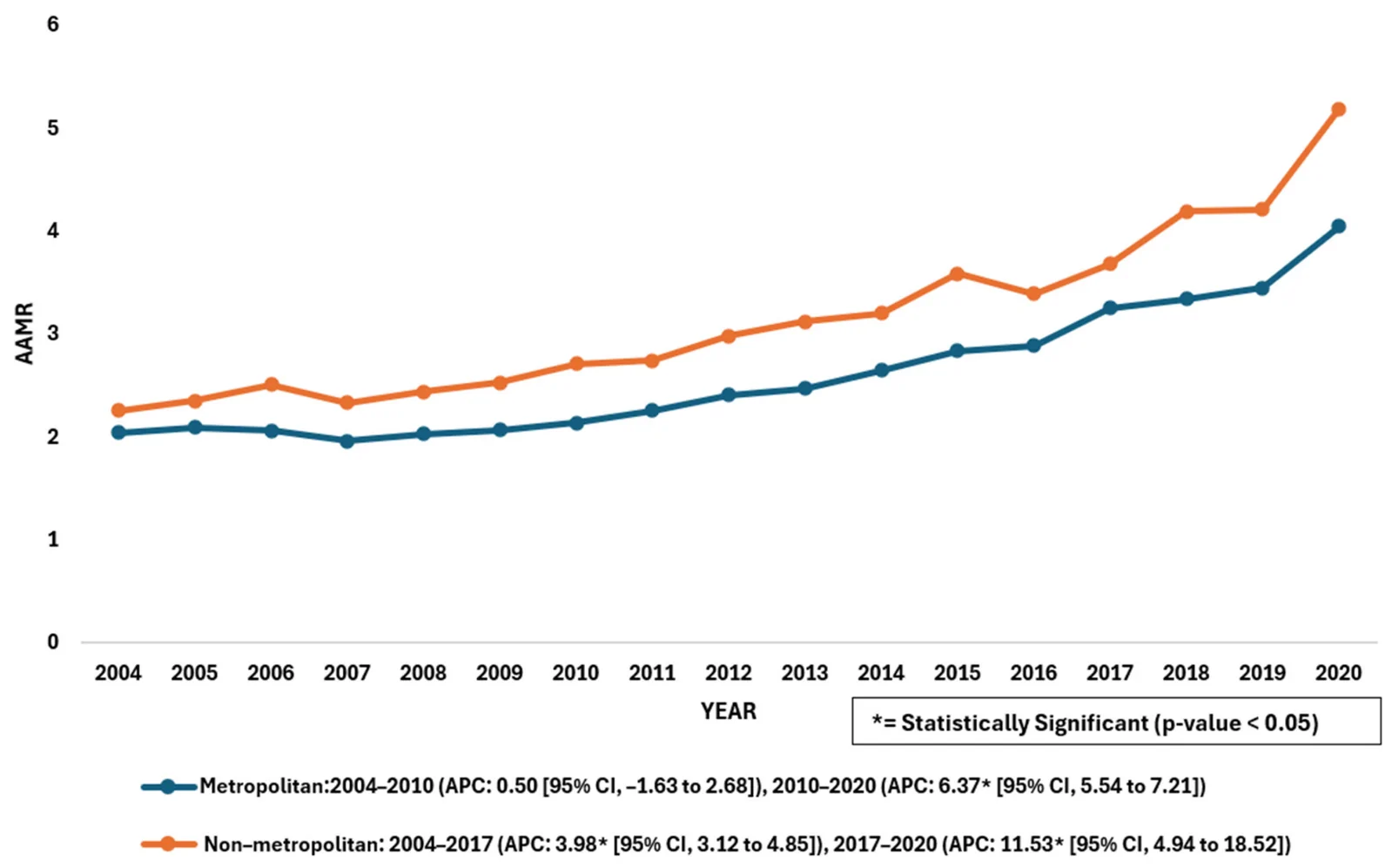
Urbanization-stratified age-adjusted mortality rates (AAMRs) for mortality involving both cardiac arrest and electrolyte and acid–base disorders among U.S. adults aged ≥25 years, 2004–2020.

For urbanization status, the mean AAMR was higher in non-metropolitan areas (3.14) than in metropolitan areas (2.59). In non-metropolitan areas, AAMR increased from 2.26 (95% CI: 2.1 to 2.42) in 2004 to 5.19 (95% CI: 4.96 to 5.42) in 2020, with an AAPC of 5.36 (95% CI: 4.11 to 6.62; *p* < 0.000001). A significant increase was observed from 2004 to 2017 (APC: 3.98; 95% CI: 3.12 to 4.85; *p* < 0.000001), followed by a further increase from 2017 to 2020 (APC: 11.53; 95% CI: 4.94 to 18.52; *p* = 0.002085). In metropolitan areas, AAMR increased from 2.04 (95% CI: 1.96 to 2.11) in 2004 to 4.05 (95% CI: 3.97 to 4.14) in 2020, with an AAPC of 4.13 (95% CI: 3.25 to 5.02; *p* < 0.000001). A non-significant increase was observed from 2004 to 2010 (APC: 0.50; 95% CI: −1.63 to 2.68; *p* = 0.619), followed by a significant increase from 2010 to 2020 (APC: 6.37; 95% CI: 5.54 to 7.21; *p* < 0.000001) (Figure 5, Supplementary Tables S5 and S8).

### 3.7. Stratified by State

From 2004 to 2020, the highest AAMR was observed in Nevada (5.99), while Illinois had the lowest (1.15). States in the 90th percentile included Nevada, Mississippi, North Dakota, South Carolina, and Connecticut, whereas the lowest 10th percentile comprised Illinois, Minnesota, Maryland, Delaware, and Virginia (Supplementary Figure S1 and Table S6).

From 2021 to 2024, Nevada remained the highest (10.65), while Maine had the lowest (1.70). The top 90th percentile included Nevada, Oregon, Louisiana, Arkansas, and Mississippi, whereas the lowest 10th percentile included Maine, the District of Columbia, Delaware, Illinois, and Michigan (Supplementary Figure S2 and Table S6).

### 3.8. Stratified by Place of Death

Place-of-death analysis revealed that the majority of deaths occurred in medical facility inpatient settings (109,772 deaths), followed by medical facility outpatient or emergency department settings (13,089 deaths), nursing homes or long-term care facilities (13,062 deaths), decedents’ homes (12,884 deaths), other locations (1979 deaths), and hospice facilities (1532 deaths). Unknown settings accounted for the remaining deaths (Supplementary Figure S3 and Table S7).

## 4. Discussion

This is a comprehensive analysis of mortality data from the publicly available CDC WONDER database, spanning from 2004 to 2024 and highlighting temporal trends in deaths coded with cardiac arrest (ICD-10 I46) and concurrent electrolyte/acid–base abnormalities (ICD-10 E87). Between 2004 and 2024, a total of 152,937 deaths were recorded, and overall age-adjusted mortality doubled, reflecting an increasing frequency of death records coded with both conditions. These findings warrant further investigation into clinical, healthcare, and demographic factors underlying these coding patterns. Although joinpoint analysis identified considerable accelerations in mortality through 2009–2021, a markedly declining trend was observed thereafter. Increasing mortality was seen across various demographic groups, but was greatest in males, adults aged ≥65 years, and Black and American Indian/Alaska Native populations, which showed the highest mean AAMRs. Disparities subject to urbanization status and geographical location also persisted, with the highest mortality in western and rural regions, highlighting the role of social determinants of health in outcomes of cardiac arrest with concurrent electrolyte and metabolic imbalances.

Our analysis identified four phases related to CA and concurrent metabolic derangement mortality trends: a stable early period (2004–2009), a sustained rise in mortality (2009–2018), a pronounced acceleration (2018–2021), and a significant decline thereafter through 2024. In-hospital cardiac arrests (IHCAs) occur in approximately 290,000 individuals annually in the United States, and the incidence of IHCAs occurring in emergency departments reportedly approaches 1.2 per 1000 visits, with elderly and men facing the highest impact [15,16]. Annual incidence of out-of-hospital CA (OHCA) also remains significant in the U.S., with studies reporting a rate of 81.3 per 100,000 person-years, 20.9 for shockable and 59.8 for non-shockable EMS-treated OHCA [17]. In addition, subsequent widespread acidosis reduces adrenoreceptor expression on cardiac cells, especially beta receptors, resulting in decreased response to circulating catecholamines and impaired left ventricular contractility, and has been associated with poorer health outcomes [18]. However, it is also worth noting that electrolyte and acid–base abnormalities remain prevalent in critically ill patients, including those with CA, but they frequently are a result of an underlying serious illness such as sepsis, renal diseases, and shock. Thus, this imbalance that is directly associated with cardiac arrest on death certificates highlights the complex nature of these terminal clinical events and multimorbidity rather than a direct causal relationship. Although our study demonstrates upward trends throughout most of the study period, this contrasts with a previous nationwide analysis reporting decreasing overall CA mortality [19]. We believe this discrepancy can be explained by different outcomes studied in our analysis, which specifically captured deaths involving both CA and concurrent metabolic abnormalities rather than CA alone. The increasing prevalence of chronic conditions, such as complicated diabetes and hypertension, hyperlipidemia, and obesity over the years, may have further exacerbated the rise in metabolic and electrolyte disturbances, as diabetes is commonly linked with hyponatremia, hyperkaliemia, hypomagnesemia, hypocalcemia, and anion-gap and hyperchloremic normal-gap acidosis seen frequently in diabetic ketoacidosis [20,21]. Chronic kidney disease is another condition commonly associated with electrolyte and acid–base abnormalities especially in sudden cardiac death cases, and may partially explain the increasing co-occurrence of these metabolic imbalances in mortality records [22]. Furthermore, according to the Cardiac Arrest Registry to Enhance Survival, 21.6% of patients with OHCA are found dead before reaching a medical facility, and many, unfortunately, do not receive bystander-aided cardiopulmonary resuscitation (CPR) and other necessary interventions known to increase survival benefit, highlighting significant gaps in prehospital OHCA care and its contributions to an overall higher CA mortality [23]. The updated American Heart Association (AHA) resuscitation guidelines have improved in-hospital cardiac arrest management through standardized ACLS (advanced cardiovascular life support) and enhanced post-resuscitation care, including extracorporeal CPR in refractory cases, early coronary intervention, postarrest therapeutic hypothermia, and advanced critical care, resulting in recent declines in mortality [24]. Significant declines in mortality observed across most demographic groups also reflect a positive shift in healthcare towards an overall reduced disease burden, potentially reflecting increased healthcare accessibility, emergency CVD care, and more effective screening and management plans.

The rapid shift toward positive trends through 2018–2021 may, in part, reflect the impact of the COVID-19 pandemic, as various studies from all over the world confirm the synchronous rise in CA incidence with COVID-19 [25]. This could be elucidated by social isolation brought on by the pandemic secondary to lockdowns, as well as reduced bystander cardiopulmonary resuscitation before EMS, lung failure due to hypoxia, compromised cardiac function and myocardial damage, and mechanical ventilation-induced injuries [25]. The SARS-CoV-2 virus leads to acute renal injury by binding to ACE2 receptors on podocytes and tubular epithelial cells, and also damages the GI mucosa, causing several GI manifestations such as diarrhea and vomiting [26]. These pathological factors may contribute to significant fluid and electrolyte imbalances in COVID-19, including hyponatremia, hypokalemia, and hypochloremia [26]. Research also confirms higher reported mortality and worse clinical outcomes in COVID-19, particularly in patients with concurrent hyponatremia, hypernatremia, and hypocalcemia [27]. Several acid–base derangements have also been associated with COVID-19 infection, with respiratory alkalosis found in 40% of patients and metabolic alkalosis in 30% [28]. Interestingly, this period exhibited the most pronounced annual percent change (APC) across the entire study interval for both genders; men displayed a higher disease burden, with males experiencing an APC of 13.10% and females an APC of 10.63%. This finding aligns with a previous meta-analysis showing an overall greater COVID-19 mortality in male cohorts as compared with females [29]. This could be partly explained by gender-based immunological variations driven by sex hormones independent of age, as estrogen may have protective effects against the infection [30].

Sex-stratified analyses revealed that males consistently experienced higher age-adjusted mortality rates than females throughout the study period, with both sexes demonstrating significant net increases overall. Males exhibited a higher average annual percent change (AAPC: 3.50%) compared with females (AAPC: 3.04%), indicating a slightly greater cumulative increase over time. This finding is consistent with a previous study, with a majority of male patients reporting poor outcomes post-OHCA and increased ICU mortality in patients with higher base deficit quartiles [31]. Research also proves greater use of diuretics and aldosterone antagonists in men, with a higher reported hyperkalemia incidence post diuretic use, although female sex has been largely associated with hypokalemia [32]. Therefore, the observed overall higher CA-related mortality in our study and increased use of diuretics in men may reflect multiple factors, including greater cardiovascular comorbidity burden, such as heart failure, a detrimental cardiac condition that is vastly associated with acute circulatory collapse, ventricular tachyarrhythmias, asystole, and contractile failure, all of which are direct precursors to sudden death [33]. These sex-based disparities could be further elucidated by the cardioprotective roles of estrogen, as well as its crucial impact on neuroendocrine regulation of normal sodium and fluid levels [34], highlighting the reason behind overall lower AAMR observed in women.

As the American elderly population grows over time, the comorbidities in the elderly have also increased, such as cardiac failure and ischemic heart disease [35], further adding to the higher baseline vulnerability observed in this age group. Older age is an unmodifiable risk factor for cardiac diseases, as research clearly shows increasing prevalence and mortality of CVDs with age, with around 82% CVD-associated deaths occurring in those aged 65 years and above [36]. Older adults, predominantly those ≥65 years, account for most hospitalizations secondary to cardiac and other causes, with 70-year-olds accounting for most ICU admissions [36,37], which may partially explain the higher mortality rates observed among individuals aged ≥65 years in the present analysis. American adults aged 60 years and above are noted to have a CKD (chronic kidney disease) prevalence of around 39% [38]. This is partly because of numerous modifiable risk factors, such as diabetes, hypertension, and obesity, as well as CVDs, alongside non-modifiable elements too, with aging being one of the most important predictors of CKD [38]. However, declining GFR is considered part of normal physiological age-related events as well, even in the absence of any disease, with a reduction of about 8 mL/min/1.73 m^2^ per decade, and aging-associated decline in glomerular function is known to start in the 30–40s years of age [39]. Therefore, the higher prevalence of CKD and declining renal function in older adults may also add to the frequent co-occurrence of electrolyte and acid/base disorders in this population; however, because the present study was based on death certificate data, it cannot determine whether these imbalances directly increased mortality or primarily reflected severe underlying illnesses. In addition, delayed recognition of worsening illness, hesitation to start aggressive treatment regimens, and atypical or asymptomatic presentation of diseases in the elderly also likely contribute to the poorer outcomes in this population.

In our analysis, although all racial groups experienced overall rising trends through 2021, Black and American Indian/Alaska Native populations were found to have the highest overall AAMR, with Asian people demonstrating the lowest mortality by 2024. This finding is in alignment with a CDC report studying racial- and sex-based IHCA mortality, where Black and Hispanic populations were generally found to suffer the most from CA-related mortality regardless of the cause [40]. The authors observed that minority groups were more likely to belong to the lower socioeconomic strata [40], highlighting a key social determinant of health that may contribute to higher mortality in these populations by limiting their ability to access medical care as frequently as needed. Moreover, because electrolyte and acid/base disturbances frequently occur in critically ill patients and are commonly recorded alongside CA, the overall higher mortality observed among the minority groups could also potentially highlight differences in the prevalence of severe underlying diseases and inequities in healthcare, as this pattern suggests not only delayed presentation and lack of acute care but also broader structural barriers faced by vulnerable populations. Several demographic studies confirm this racial disparity; prior to 2010, ACA Hispanics and American Indians/Alaska Natives were most likely not to receive medical insurance, but disparities persisted even in the later years [41]. Although widespread insurance coverage gains were observed during the COVID-19 pandemic, the uninsured rate for people under 65 years of age rapidly increased thereafter by 1.3 million, with AIAN and Hispanic individuals demonstrating the highest rate of uninsurance at 18.9% and 18.4% by 2024, respectively [41].

Rural communities consistently experienced an overall greater mortality rate across the study period in comparison with their urban counterparts. This is in contrast with the previous CDC analysis, which reported a higher CA-related mortality in urban populations with CKD, a finding that the authors attributed to metropolitan infrastructure as well as greater incidence of CVD-promoting factors in urban areas, including pollution and poor sleep [42]. However, we believe this discrepancy may reflect differences in study population and coding patterns, as our analysis included mortality data about CA co-occurring with electrolyte and acid/base disturbances, rather than CKD-associated CA alone. Moreover, the persistent rise in the AAMR observed for non-metropolitan areas in our study could be subject to reduced odds of return of spontaneous circulation, along with increased emergency medical services response times observed in rural OHCA, as compared to urban cases [43]. Residents of rural regions also face healthcare accessibility issues, where specialty care is not available to them locally, highlighting significant geographical barriers to medical aid [44]. Certain rural communities also travel longer distances to access necessary medical services, and these disadvantaged populations face considerable difficulties with health service utilization due to affordability issues [44]. Although our study has demonstrated significant regional disparities, with the west and northeast exhibiting the highest AAMRs overall, this is in contrast with the previous study, which has reported the greatest CA-associated mortality in the southern regions, with the lowest mortality in the west [19]. This apparent discrepancy may reflect regional variation in cardiac arrest outcomes, which are heavily influenced by hospital infrastructure, and resuscitation protocols, bystander cardiopulmonary resuscitation (CPR) knowledge and skills, public availability of automated external defibrillators (AEDs), and differences in healthcare accessibility and cost. This variation is further reaffirmed by prior studies showing clear regional differences in survival to discharge rates and bystander CPR post-OHCA, with the majority of the patients in the lowest quartile being Black [45], further highlighting racial disparities as well. Moreover, Dhaval Kolte et al. in their analysis found IHCA incidence in the West to be the highest amongst all U.S. census regions (3.73 per 1000 admissions), with regional variations in survival rates and hospital costs [46]. Interestingly, patients from the western and southern regions were also more likely to exhibit an electrolyte/fluid abnormality compared with those from the northeast [46]. Taken together, these findings suggest that higher mortality in the west and the northeast may reflect regional variation in healthcare access, underlying comorbidity burden, healthcare practices, and coding of concurrent conditions rather than differences in CA alone. The interstate differences, with persistently higher AAMRs in states such as Nevada and lower rates in states such as Illinois and Maine, also likely reflect variation in baseline risk factors and comorbidity patterns, as well as differences in healthcare access and quality of care. Lastly, most cardiac arrest deaths occurred in inpatient hospital facilities, followed by hospital emergency departments and outpatient facilities, likely reflecting the concentration of critically ill patients, severity of comorbid health conditions, increased burden of elderly patients, and the fact that these departments capture more complicated and acutely ill medical cases, such as cardiopulmonary arrest.

Strengths and limitations: Our study had several strengths with multiple important findings, making it a novel topic; we utilized an extensive mortality dataset using the publicly available CDC WONDER database and assessed temporal trends across various demographics and U.S. states to create a wider and more systemic understanding of not only cardiac arrest but its concurrence with electrolyte and acid–base disorders as well, especially in underrepresented demographics. Our study spanned two decades, allowing for assessment of long-term mortality trends and health inequalities. Social determinants of health, such as socioeconomic backgrounds and patient-level characteristics, discussed in our study, help reflect on population-based trends rather than simply focusing on the biology of the disease processes. Furthermore, the use of AAMRs and jointpoint modeling ensures reliable detection of trends over time. However, we used the ICD codes, which may be subject to human error and misclassification as they are entered by the physicians, resulting in underreporting. An additional limitation is that the observed increase in mortality during the COVID-19 pandemic period (2018–2021) may have been partially influenced by the broader mortality burden of COVID-19. Because severe COVID-19 can frequently culminate in cardiac arrest and is often accompanied by electrolyte and acid–base disturbances, some of the observed increase may reflect the impact of the pandemic rather than an isolated increase in cardiac arrest associated with electrolyte and acid–base disorders. Therefore, the APC estimates during the pandemic period should be interpreted with caution. Additionally, the database lacks baseline patient characteristics such as BMI and also disease-related information, including CA type, severity, treatment strategies, type of metabolic disturbance, etc., which limits our ability to assess the underlying mechanisms driving mortality trends. Causes of mortality in a multiple-cause dataset may be challenging to identify, and certain health states, particularly chronic conditions, may be reasonably underreported on death certificates. Moreover, urbanization analyses were limited to 2004–2020 due to the unavailability of county-level urbanization classifications in the CDC WONDER database for later years at the time of analysis. Moreover, because cardiac arrest may be recorded as the terminal mechanism of death and electrolyte or acid–base disorders may represent antecedent conditions, complications of critical illness, or coding practices, our findings should be interpreted as mortality involving both conditions rather than evidence of causal or temporal relationships.

## 5. Conclusions

In conclusion, this study demonstrated an overall rise in mortality involving cardiac arrest and electrolyte and acid–base disorders in the United States from 2004 to 2024, with substantial demographic and geographic disparities. Higher mortality for various underserved and minority groups, such as Black and AIAN populations and non-metropolitan areas, observed in our analysis emphasizes the need to address accessibility gaps and equity in healthcare. Mortality remained consistently higher for older age groups, males, and residents of the western regions, further highlighting the importance of targeted public health interventions and timely recognition of metabolic disturbances in patients at risk for CA. However, further research is needed to better understand mechanisms underlying these trends and the clinical significance of concurrent metabolic disorders in CA-related health outcomes.

## Data Availability

The data analyzed in this study are publicly available through the CDC WONDER Multiple Cause of Death database.

## Supplementary Materials

The following supporting information can be downloaded at https://www.mdpi.com/article/10.3390/jcm15166258/s1, Table S1: Overall and sex-stratified age-adjusted mortality rates (AAMRs) for mortality involving cardiac arrest and electrolyte and acid–base disorders, United States, 2004–2024. Table S2. Age-stratified Crude rates (CMRs) for mortality involving cardiac arrest and electrolyte and acid–base disorders, United States, 2004–2024. Table S3. Race/ethnicity-stratified age-adjusted mortality rates (AAMRs) for mortality involving cardiac arrest and electrolyte and acid–base disorders, United States, 2004–2024. Table S4. Census region–stratified age-adjusted mortality rates (AAMRs) for mortality involving cardiac arrest and electrolyte and acid–base disorders, United States, 2004–2024. Table S5. Urbanization-stratified age-adjusted mortality rates (AAMRs) for mortality involving cardiac arrest and electrolyte and acid–base disorders, United States, 2004–2020. Table S6. State-level age-adjusted mortality rates (AAMRs) for mortality involving cardiac arrest and electrolyte and acid–base disorders, United States, 2004–2024. Table S7. Distribution of place of death for mortality involving cardiac arrest and electrolyte and acid–base disorders among U.S. adults aged ≥25 years, United States, 2004–2024. Table S8. Joinpoint regression analysis showing Annual percent change (APC) and Average annual percent change (AAPC) for mortality involving cardiac arrest and electrolyte and acid–base disorders across all demographic and geographic subgroups, United States, 2004–2024. Table S9. Age-adjusted mortality trends according to individual ICD-10 E87 electrolyte and acid–base disorder sub-codes among deaths involving cardiac arrest in U.S. adults aged ≥25 years, United States, 2004–2024. Figure S1. State-level age-adjusted mortality rates (AAMRs) for mortality involving cardiac arrest and electrolyte and acid–base disorders among U.S. adults aged ≥25 years, 2004–2020. Figure S2. State-level age-adjusted mortality rates (AAMRs) for mortality involving cardiac arrest and electrolyte and acid–base disorders among U.S. adults aged ≥25 years, 2021–2024. Figure S3. Place of death distribution for mortality involving cardiac arrest and electrolyte and acid–base disorders among U.S. adults aged ≥25 years, United States, 2004–2024. Figure S4. Age-adjusted mortality rates according to individual electrolyte and acid–base disorder diagnoses (ICD-10 E87 sub-codes) among deaths involving cardiac arrest in U.S. adults aged ≥25 years, United States, 2004–2024.
