# Supplementary material for: Mortality Trends Involving Cardiac Arrest and Fluid, Electrolyte, and Acid–Base Disorders in U.S. Adults, 2004–2024
Source: J Clin Med. 2026 Aug 13;15(16):6258. doi: 10.3390/jcm15166258 (PMC13513112; doi:10.3390/jcm15166258)
Supplement: Supplementary file 1 [file jcm-15-06258-s001.zip › jcm-4451683-supplementary.pdf]

Supplementary Table S1. Overall and sex-stratified age-adjusted mortality rates (AAMRs) for mortality involving cardiac arrest and electrolyte and acid–base disorders, United States, 2004–2024.

Year	Age Adjusted Mortality Rate (95% Confidence Interval)		
	Overall	Female	Male
**2004**	2.06 (2.00 , 2.13)	1.90 (1.82 , 1.98)	2.26 (2.15 , 2.37)
**2005**	2.12 (2.06 , 2.19)	2.03 (1.95 , 2.12)	2.26 (2.16 , 2.37)
**2006**	2.12 (2.06 , 2.19)	2.03 (1.95 , 2.11)	2.22 (2.12 , 2.32)
**2007**	2.03 (1.97 , 2.09)	1.89 (1.81 , 1.97)	2.19 (2.09 , 2.29)
**2008**	2.10 (2.04 , 2.17)	1.94 (1.86 , 2.01)	2.32 (2.22 , 2.42)
**2009**	2.15 (2.09 , 2.21)	1.94 (1.86 , 2.02)	2.41 (2.30 , 2.51)
**2010**	2.27 (2.21 , 2.33)	2.11 (2.02 , 2.19)	2.44 (2.34 , 2.54)
**2011**	2.34 (2.27 , 2.40)	2.15 (2.06 , 2.23)	2.59 (2.49 , 2.70)
**2012**	2.48 (2.42 , 2.55)	2.25 (2.17 , 2.33)	2.77 (2.66 , 2.88)
**2013**	2.59 (2.52 , 2.65)	2.33 (2.24 , 2.41)	2.88 (2.78 , 2.99)
**2014**	2.74 (2.67 , 2.81)	2.47 (2.38 , 2.55)	3.03 (2.92 , 3.14)
**2015**	2.97 (2.90 , 3.04)	2.66 (2.57 , 2.75)	3.32 (3.21 , 3.43)
**2016**	2.97 (2.90 , 3.04)	2.62 (2.53 , 2.71)	3.40 (3.29 , 3.51)
**2017**	3.30 (3.23 , 3.37)	2.99 (2.90 , 3.08)	3.69 (3.57 , 3.80)
**2018**	3.46 (3.38 , 3.53)	3.16 (3.06 , 3.25)	3.85 (3.74 , 3.97)
**2019**	3.55 (3.48 , 3.62)	3.22 (3.12 , 3.31)	3.99 (3.88 , 4.11)
**2020**	4.22 (4.14 , 4.30)	3.66 (3.56 , 3.76)	4.82 (4.70 , 4.95)
**2021**	4.79 (4.71 , 4.88)	4.20 (4.09 , 4.31)	5.49 (5.35 , 5.63)
**2022**	4.50 (4.42 , 4.59)	3.93 (3.83 , 4.03)	5.18 (5.05 , 5.31)
**2023**	4.24 (4.16 , 4.32)	3.72 (3.62 , 3.82)	4.78 (4.66 , 4.91)
**2024**	4.08 (4.00 , 4.16)	3.61 (3.52 , 3.71)	4.55 (4.43 , 4.67)

Supplementary Table S2. Age-stratified Crude rates (CMRs) for mortality involving cardiac arrest and electrolyte and acid–base disorders, United States, 2004–2024.

Year	Crude Rate (95% Confidence Interval)				
	25-44 Years	45-64 Years	65-74 Years	75-84 Years	85+ Years
2004	0.24 (0.21 , 0.28)	1.18 (1.10 , 1.26)	3.67 (3.39 , 3.94)	8.59 (8.09 , 9.10)	24.11 (22.68 , 25.54)
2005	0.25 (0.22 , 0.29)	1.27 (1.19 , 1.35)	3.54 (3.27 , 3.81)	9.07 (8.55 , 9.59)	25.38 (23.94 , 26.82)
2006	0.24 (0.21 , 0.28)	1.36 (1.28 , 1.45)	3.75 (3.48 , 4.02)	8.56 (8.06 , 9.06)	24.35 (22.97 , 25.74)
2007	0.25 (0.22 , 0.29)	1.21 (1.13 , 1.29)	3.42 (3.16 , 3.67)	8.53 (8.03 , 9.04)	23.16 (21.83 , 24.49)

2008	0.25 (0.22 , 0.29)	1.30 (1.22 , 1.38)	3.83 (3.57 , 4.10)	9.04 (8.52 , 9.55)	22.77 (21.47 , 24.07)
2009	0.27 (0.24 , 0.31)	1.38 (1.30 , 1.46)	3.84 (3.58 , 4.11)	8.90 (8.39 , 9.41)	23.33 (22.03 , 24.62)
2010	0.25 (0.21 , 0.28)	1.51 (1.42 , 1.59)	4.14 (3.87 , 4.41)	9.07 (8.55 , 9.58)	24.94 (23.62 , 26.26)
2011	0.26 (0.22 , 0.29)	1.44 (1.35 , 1.52)	4.64 (4.36 , 4.93)	10.19 (9.65 , 10.74)	24.21 (22.94 , 25.48)
2012	0.32 (0.28 , 0.36)	1.70 (1.62 , 1.79)	4.64 (4.36 , 4.91)	10.32 (9.78 , 10.87)	25.22 (23.94 , 26.51)
2013	0.33 (0.29 , 0.36)	1.91 (1.82 , 2.00)	4.67 (4.40 , 4.93)	10.58 (10.03 , 11.13)	24.63 (23.38 , 25.88)
2014	0.39 (0.35 , 0.43)	1.95 (1.86 , 2.05)	5.03 (4.76 , 5.30)	11.39 (10.83 , 11.96)	25.51 (24.25 , 26.77)
2015	0.34 (0.31 , 0.38)	2.31 (2.21 , 2.41)	5.87 (5.58 , 6.15)	11.63 (11.06 , 12.19)	26.98 (25.69 , 28.26)
2016	0.36 (0.32 , 0.40)	2.34 (2.24 , 2.45)	5.77 (5.50 , 6.05)	11.60 (11.04 , 12.16)	27.63 (26.34 , 28.92)
2017	0.49 (0.45 , 0.54)	2.55 (2.44 , 2.66)	6.53 (6.23 , 6.82)	12.46 (11.89 , 13.03)	30.19 (28.85 , 31.53)
2018	0.53 (0.48 , 0.58)	2.73 (2.62 , 2.84)	6.74 (6.44 , 7.03)	12.99 (12.42 , 13.55)	31.43 (30.07 , 32.79)
2019	0.52 (0.47 , 0.57)	2.84 (2.73 , 2.96)	7.47 (7.17 , 7.77)	13.48 (12.91 , 14.05)	30.05 (28.73 , 31.38)
2020	0.68 (0.62 , 0.73)	3.58 (3.45 , 3.71)	8.27 (7.96 , 8.59)	15.61 (15.01 , 16.21)	34.54 (33.13 , 35.95)
2021	0.82 (0.76 , 0.88)	3.95 (3.81 , 4.08)	9.12 (8.80 , 9.45)	18.20 (17.55 , 18.86)	39.36 (37.77 , 40.95)
2022	0.70 (0.65 , 0.76)	3.82 (3.69 , 3.95)	9.01 (8.69 , 9.34)	16.99 (16.38 , 17.60)	35.22 (33.77 , 36.66)
2023	0.71 (0.66 , 0.77)	3.51 (3.38 , 3.64)	8.49 (8.18 , 8.79)	15.33 (14.76 , 15.90)	35.64 (34.16 , 37.13)
2024	0.64 (0.59 , 0.69)	3.43 (3.30 , 3.55)	8.00 (7.71 , 8.30)	15.40 (14.85 , 15.96)	33.30 (31.89 , 34.71)

Supplementary Table S3. Race/ethnicity-stratified age-adjusted mortality rates (AAMRs) for mortality involving cardiac arrest and electrolyte and acid–base disorders, United States, 2004–2024.

Year	Age Adjusted Mortality Rate (95% Confidence Interval)				
	NH Black or African American	NH White	Hispanic or Latino	NH Asian or Pacific Islanders	NH American Indian or Alaska Native

**2004**	3.84 (3.54 , 4.14)	1.84 (1.77 , 1.91)	2.39 (2.10 , 2.69)	2.17 (1.77 , 2.58)	3.22 (2.12 , 4.68)
**2005**	4.15 (3.84 , 4.46)	1.88 (1.81 , 1.95)	2.48 (2.19 , 2.78)	2.31 (1.90 , 2.72)	2.12 (1.31 , 3.24)
**2006**	3.77 (3.48 , 4.07)	1.89 (1.82 , 1.95)	2.76 (2.46 , 3.06)	2.55 (2.13 , 2.97)	3.13 (2.10 , 4.49)
**2007**	3.61 (3.32 , 3.89)	1.80 (1.73 , 1.86)	2.22 (1.95 , 2.48)	2.26 (1.89 , 2.64)	3.07 (2.04 , 4.43)
**2008**	3.56 (3.29 , 3.84)	1.92 (1.85 , 1.98)	2.25 (2.00 , 2.51)	2.11 (1.75 , 2.46)	3.08 (2.10 , 4.34)
**2009**	3.57 (3.30 , 3.85)	1.93 (1.86 , 2.00)	2.29 (2.04 , 2.54)	2.40 (2.04 , 2.76)	3.14 (2.13 , 4.46)
**2010**	3.68 (3.41 , 3.96)	2.05 (1.98 , 2.12)	2.50 (2.24 , 2.76)	1.89 (1.57 , 2.21)	4.77 (3.53 , 6.31)
**2011**	4.16 (3.87 , 4.44)	2.09 (2.02 , 2.16)	2.71 (2.45 , 2.97)	2.32 (1.98 , 2.66)	4.19 (3.08 , 5.58)
**2012**	4.24 (3.95 , 4.52)	2.27 (2.20 , 2.34)	2.52 (2.28 , 2.76)	2.20 (1.88 , 2.52)	4.48 (3.35 , 5.86)
**2013**	4.54 (4.25 , 4.82)	2.29 (2.22 , 2.37)	2.82 (2.57 , 3.07)	2.05 (1.75 , 2.35)	4.00 (3.02 , 5.20)
**2014**	4.69 (4.40 , 4.99)	2.48 (2.40 , 2.55)	3.09 (2.84 , 3.34)	2.03 (1.74 , 2.31)	4.44 (3.38 , 5.72)
**2015**	5.18 (4.88 , 5.48)	2.63 (2.56 , 2.71)	3.25 (3.01 , 3.50)	2.17 (1.89 , 2.45)	5.65 (4.45 , 7.07)
**2016**	5.26 (4.96 , 5.55)	2.66 (2.58 , 2.73)	3.28 (3.03 , 3.52)	2.25 (1.97 , 2.52)	6.01 (4.81 , 7.42)
**2017**	5.56 (5.26 , 5.86)	2.98 (2.90 , 3.06)	3.48 (3.23 , 3.72)	2.70 (2.41 , 3.00)	5.74 (4.59 , 7.09)
**2018**	5.75 (5.44 , 6.05)	3.16 (3.08 , 3.24)	3.47 (3.23 , 3.71)	2.63 (2.34 , 2.92)	5.86 (4.70 , 7.21)
**2019**	6.13 (5.82 , 6.44)	3.21 (3.13 , 3.29)	3.71 (3.46 , 3.95)	2.73 (2.44 , 3.02)	6.43 (5.17 , 7.69)
**2020**	7.54 (7.20 , 7.88)	3.62 (3.54 , 3.71)	4.99 (4.72 , 5.26)	3.49 (3.17 , 3.80)	7.30 (5.99 , 8.60)
**2021**	8.47 (8.11 , 8.84)	4.17 (4.08 , 4.27)	5.54 (5.26 , 5.84)	3.91 (3.58 , 4.27)	7.01 (5.75 , 8.48)
**2022**	7.92 (7.58 , 8.28)	3.95 (3.86 , 4.04)	5.04 (4.78 , 5.32)	3.75 (3.44 , 4.08)	7.74 (6.42 , 9.28)
**2023**	7.52 (7.19 , 7.86)	3.74 (3.65 , 3.83)	4.82 (4.57 , 5.08)	3.17 (2.89 , 3.47)	7.35 (6.09 , 8.81)
**2024**	7.46 (7.14 , 7.80)	3.51 (3.42 , 3.60)	4.61 (4.37 , 4.86)	3.19 (2.92 , 3.48)	6.64 (5.47 , 8.02)

Supplementary Table S4. Census region–stratified age-adjusted mortality rates (AAMRs) for mortality involving cardiac arrest and electrolyte and acid–base disorders, United States, 2004–2024.

Year	Age Adjusted Mortality Rate (95% Confidence Interval)			
	Northeast	Midwest	South	West
**2004**	2.41 (2.26 , 2.57)	1.47 (1.36 , 1.59)	1.93 (1.83 , 2.04)	2.52 (2.37 , 2.68)
**2005**	2.57 (2.42 , 2.73)	1.58 (1.46 , 1.70)	2.10 (1.99 , 2.20)	2.41 (2.26 , 2.56)
**2006**	2.46 (2.31 , 2.61)	1.50 (1.39 , 1.61)	2.08 (1.97 , 2.19)	2.60 (2.44 , 2.76)
**2007**	2.40 (2.25 , 2.55)	1.40 (1.29 , 1.50)	1.97 (1.87 , 2.07)	2.41 (2.26 , 2.55)
**2008**	2.57 (2.42 , 2.72)	1.43 (1.32 , 1.54)	2.09 (1.98 , 2.19)	2.45 (2.30 , 2.59)
**2009**	2.56 (2.41 , 2.72)	1.51 (1.40 , 1.62)	2.19 (2.08 , 2.29)	2.38 (2.23 , 2.52)
**2010**	2.71 (2.55 , 2.87)	1.50 (1.39 , 1.61)	2.31 (2.20 , 2.42)	2.52 (2.38 , 2.67)
**2011**	2.78 (2.62 , 2.94)	1.75 (1.63 , 1.87)	2.28 (2.17 , 2.38)	2.69 (2.54 , 2.84)
**2012**	2.84 (2.68 , 3.00)	1.99 (1.86 , 2.12)	2.50 (2.39 , 2.61)	2.70 (2.56 , 2.85)
**2013**	2.88 (2.73 , 3.04)	1.93 (1.81 , 2.05)	2.66 (2.54 , 2.77)	2.80 (2.65 , 2.95)
**2014**	3.21 (3.04 , 3.38)	1.97 (1.85 , 2.09)	2.80 (2.69 , 2.91)	2.90 (2.75 , 3.05)
**2015**	3.28 (3.11 , 3.45)	2.28 (2.15 , 2.41)	3.02 (2.91 , 3.14)	3.19 (3.04 , 3.35)
**2016**	3.51 (3.34 , 3.68)	2.25 (2.12 , 2.38)	2.91 (2.80 , 3.02)	3.28 (3.13 , 3.43)
**2017**	3.66 (3.48 , 3.84)	2.47 (2.33 , 2.60)	3.17 (3.05 , 3.28)	3.99 (3.83 , 4.16)
**2018**	3.68 (3.50 , 3.85)	2.58 (2.44 , 2.71)	3.35 (3.24 , 3.47)	4.31 (4.14 , 4.49)
**2019**	3.83 (3.66 , 4.01)	2.89 (2.75 , 3.03)	3.31 (3.19 , 3.43)	4.41 (4.24 , 4.59)
**2020**	4.82 (4.62 , 5.01)	3.24 (3.09 , 3.39)	3.93 (3.80 , 4.05)	5.11 (4.93 , 5.30)
**2021**	5.21 (5.00 , 5.42)	3.19 (3.04 , 3.35)	4.84 (4.70 , 4.98)	5.85 (5.65 , 6.05)
**2022**	5.05 (4.85 , 5.25)	3.12 (2.98 , 3.28)	4.24 (4.11 , 4.37)	5.68 (5.49 , 5.88)
**2023**	4.71 (4.52 , 4.91)	2.98 (2.83 , 3.13)	3.99 (3.87 , 4.12)	5.34 (5.16 , 5.53)
**2024**	4.83 (4.64 , 5.03)	2.90 (2.76 , 3.04)	3.85 (3.73 , 3.98)	4.83 (4.66 , 5.00)

Supplementary Table S5. Urbanization-stratified age-adjusted mortality rates (AAMRs) for mortality involving cardiac arrest and electrolyte and acid–base disorders, United States, 2004–2020.

Year	Age Adjusted Mortality Rate (95% Confidence Interval)	
	Metropolitan	Non-Metropolitan
**2004**	2.04 (1.96 , 2.11)	2.26 (2.10 , 2.41)
**2005**	2.09 (2.02 , 2.16)	2.35 (2.19 , 2.52)
**2006**	2.06 (1.99 , 2.13)	2.51 (2.35 , 2.68)
**2007**	1.96 (1.89 , 2.03)	2.33 (2.17 , 2.49)
**2008**	2.03 (1.96 , 2.10)	2.44 (2.28 , 2.60)

**2009**	2.07 (2.00 , 2.13)	2.53 (2.36 , 2.69)
**2010**	2.14 (2.07 , 2.21)	2.71 (2.54 , 2.88)
**2011**	2.26 (2.19 , 2.33)	2.74 (2.57 , 2.91)
**2012**	2.41 (2.34 , 2.48)	2.98 (2.81 , 3.16)
**2013**	2.47 (2.40 , 2.54)	3.12 (2.94 , 3.30)
**2014**	2.65 (2.57 , 2.72)	3.20 (3.02 , 3.38)
**2015**	2.84 (2.77 , 2.92)	3.59 (3.40 , 3.78)
**2016**	2.89 (2.81 , 2.96)	3.39 (3.20 , 3.57)
**2017**	3.25 (3.17 , 3.32)	3.68 (3.49 , 3.88)
**2018**	3.34 (3.26 , 3.42)	4.19 (3.98 , 4.39)
**2019**	3.45 (3.37 , 3.53)	4.21 (4.01 , 4.42)
**2020**	4.05 (3.97 , 4.14)	5.19 (4.96 , 5.42)

Supplementary Table S6. State-level age-adjusted mortality rates (AAMRs) for mortality involving cardiac arrest and electrolyte and acid–base disorders, United States, 2004–2024.

**State**	**Age Adjusted Mortality Rate (95% Confidence Interval)**	
	**2004-2020**	**2021-2024**
**Alabama**	3.03 (2.89 , 3.17)	4.71 (4.37 , 5.07)
**Alaska**	1.96 (1.58 , 2.35)	4.69 (3.74 , 5.84)
**Arizona**	3.05 (2.93 , 3.17)	4.31 (4.05 , 4.59)
**Arkansas**	3.35 (3.16 , 3.53)	7.07 (6.54 , 7.64)
**California**	3.58 (3.52 , 3.64)	5.55 (5.42 , 5.69)
**Colorado**	1.80 (1.68 , 1.91)	3.58 (3.30 , 3.88)
**Connecticut**	4.00 (3.82 , 4.17)	3.04 (2.73 , 3.37)
**Delaware**	1.48 (1.26 , 1.70)	2.21 (1.76 , 2.78)
**District of Columbia**	2.69 (2.30 , 3.09)	2.11 (1.49 , 2.93)
**Florida**	2.03 (1.98 , 2.09)	3.35 (3.23 , 3.48)
**Georgia**	3.13 (3.02 , 3.24)	4.29 (4.06 , 4.53)
**Hawaii**	3.12 (2.87 , 3.38)	3.93 (3.40 , 4.53)
**Idaho**	2.04 (1.83 , 2.25)	2.72 (2.31 , 3.19)
**Illinois**	1.15 (1.09 , 1.20)	2.25 (2.10 , 2.40)
**Indiana**	2.48 (2.37 , 2.59)	2.88 (2.65 , 3.13)
**Iowa**	2.44 (2.28 , 2.59)	4.03 (3.65 , 4.45)
**Kansas**	2.55 (2.39 , 2.72)	4.64 (4.20 , 5.11)
**Kentucky**	2.76 (2.62 , 2.91)	4.88 (4.52 , 5.27)
**Louisiana**	1.92 (1.80 , 2.04)	7.41 (6.96 , 7.88)
**Maine**	1.54 (1.36 , 1.72)	1.70 (1.36 , 2.12)
**Maryland**	1.40 (1.31 , 1.49)	3.71 (3.44 , 3.99)
**Massachusetts**	2.95 (2.84 , 3.07)	6.33 (6.01 , 6.66)
**Michigan**	1.59 (1.52 , 1.66)	2.48 (2.31 , 2.66)

**Minnesota**	1.32 (1.23 , 1.41)	2.85 (2.61 , 3.12)
**Mississippi**	5.01 (4.77 , 5.24)	6.47 (5.95 , 7.03)
**Missouri**	1.84 (1.74 , 1.93)	3.38 (3.13 , 3.66)
**Montana**	1.74 (1.51 , 1.97)	3.49 (2.90 , 4.19)
**Nebraska**	3.12 (2.90 , 3.35)	5.29 (4.71 , 5.92)
**Nevada**	5.99 (5.71 , 6.27)	10.65 (10.01 , 11.33)
**New Hampshire**	1.94 (1.74 , 2.15)	2.74 (2.29 , 3.26)
**New Jersey**	2.87 (2.77 , 2.97)	5.97 (5.70 , 6.25)
**New Mexico**	2.80 (2.59 , 3.00)	4.54 (4.04 , 5.10)
**New York**	3.83 (3.76 , 3.91)	5.44 (5.26 , 5.62)
**North Carolina**	2.47 (2.38 , 2.56)	3.61 (3.41 , 3.83)
**North Dakota**	4.14 (3.72 , 4.56)	4.87 (4.01 , 5.88)
**Ohio**	3.10 (3.01 , 3.19)	3.28 (3.10 , 3.47)
**Oklahoma**	2.70 (2.54 , 2.85)	3.12 (2.81 , 3.46)
**Oregon**	2.61 (2.47 , 2.75)	8.21 (7.74 , 8.71)
**Pennsylvania**	2.41 (2.33 , 2.48)	4.26 (4.07 , 4.46)
**Rhode Island**	3.86 (3.54 , 4.18)	3.30 (2.74 , 3.97)
**South Carolina**	4.11 (3.95 , 4.28)	5.06 (4.72 , 5.42)
**South Dakota**	2.61 (2.30 , 2.92)	5.42 (4.57 , 6.39)
**Tennessee**	3.65 (3.52 , 3.79)	5.27 (4.97 , 5.59)
**Texas**	3.13 (3.06 , 3.19)	4.59 (4.44 , 4.74)
**Utah**	2.19 (2.00 , 2.37)	6.27 (5.71 , 6.87)
**Vermont**	1.99 (1.69 , 2.29)	3.79 (3.05 , 4.71)
**Virginia**	1.48 (1.41 , 1.56)	2.63 (2.44 , 2.83)
**Washington**	2.79 (2.67 , 2.91)	4.97 (4.69 , 5.26)
**West Virginia**	3.70 (3.46 , 3.93)	3.09 (2.66 , 3.58)
**Wisconsin**	1.48 (1.39 , 1.57)	2.62 (2.40 , 2.86)
**Wyoming**	1.50 (1.20 , 1.80)	3.55 (2.73 , 4.59)

Supplementary Table S7. Distribution of place of death for mortality involving cardiac arrest and electrolyte and acid–base disorders among U.S. adults aged ≥25 years, United States, 2004–2024.

Place of Death	Deaths 2004-2020	Deaths 2021-2024	Total Deaths 2004-2024	Total Death Percentage (%)
**Medical Facility - Inpatient**	74456	35316	109772	71.8
**Medical Facility - Outpatient or ER**	9046	4043	13089	8.6
**Medical Facility - Dead on Arrival**	222	51	273	0.2

**Decedent's home**	8830	4054	12884	8.4
**Hospice facility**	810	722	1532	1.0
**Nursing home/long term care**	10503	2559	13062	8.5
**Other**	1376	603	1979	1.3
**Place of death unknown**	344	Suppressed	344	0.2
**Medical Facility - Status unknown**	Missing	0	0	0

Supplementary Table S8. Joinpoint regression analysis showing Annual percent change (APC) and Average annual percent change (AAPC) for mortality involving cardiac arrest and electrolyte and acid–base disorders across all demographic and geographic subgroups, United States, 2004–2024.

Cohort	Year Range	APC (95% CI)	P-value	AAPC (95% Confidence Interval)	P-Value
**Overall**	2004-2009	0.5156 (-0.9766 to 2.0303)	0.46131	3.4178* (2.5805, 4.2619)	< 0.000001
	2009-2018	5.2514* (4.574 to 5.9333)	< 0.000001		
	2018-2021	11.9307* (6.6565 to 17.4657)	0.0004		
	2021-2024	-4.9513* (-6.9876 to -2.8705)	0.00039		
			**Gender**		
**Female**	2004-2009	-0.2387 (-2.7748 to 2.3635)	0.8403	3.0413* (1.6386, 4.4632)	0.000018
	2009-2018	5.0321* (3.9326 to 6.1433)	0.000001		
	2018-2021	10.6321* (2.0049 to 19.9889)	0.01969		
	2021-2024	-4.3635* (-7.8254 to -0.7715)	0.02246		
**Male**	2004-2007	-1.7426 (-5.2632 to 1.9087)	0.30825	3.5030* (2.5955, 4.4185)	< 0.000001
	2007-2018	5.1517* (4.6464 to 5.6595)	< 0.000001		
	2018-2021	13.1011* (7.7655 to 18.7009)	0.00021		
	2021-2024	-5.8412* (-7.8735 to -3.764)	0.00011		
			**Age Group**		

**25-44 Years**	2004-2016	4.7683* (2.8743 to 6.6972)	0.000099		
	2016-2021	15.3614* (8.7345 to 22.3921)	0.000166		
	2021-2024	-6.2212 (-15.1478 to 3.6447)	0.188698	5.5526*(3.3033,7.8508)	0.000001
**45-64 Years**	2004-2011	3.2156* (0.5924 to 5.9072)	0.019783		
	2011-2021	9.7874* (8.2757 to 11.3203)	< 0.00000		
	2021-2024	-3.2592 (-8.6701 to 2.4722)	0.235567	5.4214*(4.0648,6.7956)	<0.00000
**65-74 Years**	2004-2007	-1.9101 (-9.7526 to 6.6139)	0.617285		
	2007-2018	6.1453* (5.0909 to 7.2103)	< 0.00000		
	2018-2021	11.2239* (1.9491 to 21.3424)	0.021482		
	2021-2024	-4.3344* (-8.1132 to -0.4002)	0.034266	3.9997*(2.2007,5.8304)	0.000011
**75-84 Years**	2004-2007	-0.9566 (-6.8778 to 5.3412)	0.735482		
	2007-2018	3.6662* (2.8034 to 4.5363)	0.000002		
	2018-2021	11.6692* (2.6534 to 21.4768)	0.015267		
	2021-2024	-5.1836* (-8.6305 to -1.6066)	0.009449	2.7299*(1.1845,4.2989)	0.000497
**85+ Years**	2004-2014	0.4476 (-0.5699 to 1.4755)	0.360628		
	2014-2021	5.8493* (3.961 to 7.7719)	0.000012		
	2021-2024	-3.1356 (-7.8597 to 1.8307)	0.191911	1.7502*(0.7362,2.7743)	0.000685
			**Census Region**		
**Northeast**	2004-2009	0.8608 (-1.6625 to 3.4489)	0.46836	3.3497* (1.9851, 4.7326)	0.000001
	2009-2018	4.1952* (3.1143 to 5.2875)	0.000005		
	2018-2021	11.9933* (3.5723 to 21.0991)	0.00904		
	2021-2024	-3.0704 (-6.4707 to 0.4535)	0.0803		
**Midwest**	2004-2008	-2.0381 (-7.8015 to 4.0856)	0.47618	3.2003* (1.7272, 4.6948)	0.000018
	2008-2021	6.6046* (5.713 to 7.5038)	< 0.000001		
	2021-2024	-3.8901 (-9.2561 to 1.7932)	0.15956		
**South**	2004-2018	3.9360* (3.304 to 4.5719)	< 0.000001	3.6274* (2.0903, 5.1877)	0.000003

	2018-2021	12.8906* (2.5912 to 24.224)	0.01692		
	2021-2024	-6.1868* (-10.3861 to -1.7907)	0.00999		
**West**	2004-2014	1.5929* (0.3966 to 2.8033)	0.01283	3.7529* (2.7234, 4.7928)	< 0.000001
	2014-2021	11.0364* (9.051 to 13.0579)	< 0.000001		
	2021-2024	-5.0036* (-9.1981 to -0.6154)	0.0289		
		**Race**			
**Hispanic or Latino**	2004-2008	-2.6249 (-8.9433 to 4.1319)	0.39774	2.9370* (0.8609, 5.0558)	0.00536
	2008-2018	4.3679* (2.8858 to 5.8713)	0.000056		
	2018-2021	15.9808* (4.2683 to 29.009)	0.01119		
	2021-2024	-6.0415* (-10.3815 to -1.4913)	0.01489		
**NH American Indian or Alaska Native**	2004-2022	6.0979* (4.9785 to 7.2293)	< 0.000001	4.6661* (2.3736, 7.0099)	0.000054
	2022-2024	-7.3814 (-25.6486 to 15.3738)	0.47006		
**NH Asian or Pacific Islander**	2004-2015	-1.2142 (-3.1432 to 0.7532)	0.20372	1.4574 (-0.4033, 3.3528)	0.125505
	2015-2021	10.7750* (6.137 to 15.6156)	0.00018		
	2021-2024	-6.1439 (-13.1142 to 1.3855)	0.09927		
**NH Black or African American**	2004-2008	-3.4449 (-6.8547 to 0.0898)	0.05494	3.0465* (1.7765, 4.3324)	0.000002
	2008-2018	5.1060* (4.219 to 6.0005)	< 0.000001		
	2018-2021	13.1212* (5.5783 to 21.203)	0.0026		
	2021-2024	-4.1586* (-7.1068 to -1.1169)	0.0127		
**NH White**	2004-2009	0.7889 (-0.9477 to 2.556)	0.3375	3.3164* (2.3082, 4.3345)	< 0.000001
	2009-2018	5.2897* (4.4555 to 6.1305)	< 0.000001		
	2018-2021	10.2525* (4.1302 to 16.7347)	0.00345		
	2021-2024	-4.6705* (-7.4109 to -1.8491)	0.00443		
		**Urbanization**			

**Metropolitan**	2004-2010	0.5029 (-1.6255 to 2.6774)	0.61888	4.1312* (3.2528, 5.017)	< 0.000001
	2010-2020	6.3707* (5.536 to 7.212)	< 0.000001		
**Non-metropolitan**	2004-2017	3.9818* (3.1185 to 4.8522)	< 0.000001	5.3562* (4.1064, 6.621)	< 0.000001
	2017-2020	11.5252* (4.9427 to 18.5205)	0.00209		
**Sensitivity Analysis**					
**E87.0**	2004-2018	4.4092* (3.0576 to 5.7786)	0.000007	3.8707*(1.1355–6.6799)	0.005283
	2018-2021	20.9064* (1.9456 to 43.3937)	0.031821		
	2021-2024	-12.8920* (-19.205 to -6.0858)	0.00162		
**E87.1**	2004-2018	6.3409* (5.3105 to 7.3814)	< 0.00000	6.1590*(4.3986–7.9492)	<0.00000
	2018-2021	16.1940* (4.6152 to 29.0543)	0.008629		
	2021-2024	-3.7811 (-8.0055 to 0.6374)	0.086465		
**E87.2**	2004-2009	0.4415 (-2.6592 to 3.6409)	0.76633	4.9815*(3.9814–5.9912)	<0.00000
	2009-2021	8.5240* (7.8381 to 9.2142)	< 0.00000		
	2021-2024	-1.0401 (-4.888 to 2.9636)	0.578725		
**E87.5**	2004-2018	3.2093* (2.4216 to 4.003)	0.000001	2.8681*(1.0515–4.7173)	0.001867
	2018-2021	12.7938* (0.6162 to 26.4453)	0.040377		
	2021-2024	-7.6229* (-12.3931 to -2.593)	0.006565		
**E87.6**	2004-2014	2.4957 (-0.4628 to 5.542)	0.092138	5.1164*(2.9244–7.3552)	0.000003
	2014-2021	10.9272* (6.5991 to 15.4309)	0.000082		
	2021-2024	0.855 (-6.5568 to 8.8548)	0.813334		
**E87.8**	2004-2016	-1.3756* (-2.3328 to -0.4091)	0.008976	−0.0791 (−1.4333–1.2937)	0.909519
	2016-2021	10.6254* (6.2413 to 15.1904)	0.000122		
	2021-2024	-11.1451* (-16.584 to -5.3516)	0.001399		

Abbreviations: CI, Confidence Interval; APC, Annual Percentage Change; AAPC, Average Annual Percentage Change

*** Indicates that the APC significantly differs from zero at the alpha = 0.05 level.**

Supplementary Table S9: Age-adjusted mortality trends according to individual ICD-10 E87 electrolyte and acid–base disorder sub-codes among deaths involving cardiac arrest in U.S. adults aged ≥25 years, United States, 2004–2024.

Year	Age Adjusted Mortality Rate (95% Confidence Interval					
	E87.0 (Hyperosmolality and hypernatremia)	E87.1 (Hypo-osmolality and hyponatremia)	E87.2 (Acidosis)	E87.5 (Hyperkalemia)	E87.6 (Hypokalemia)	E87.8 (Other disorders of electrolyte and fluid balance, not elsewhere classified)
2004	0.12 (0.10 , 0.13)	0.11 (0.10 , 0.13)	0.70 (0.66 , 0.74)	0.60 (0.57 , 0.64)	0.10 (0.09 , 0.12)	0.39 (0.36 , 0.42)
2005	0.09 (0.08 , 0.11)	0.12 (0.10 , 0.13)	0.76 (0.72 , 0.80)	0.67 (0.64 , 0.71)	0.11 (0.09 , 0.12)	0.42 (0.39 , 0.45)
2006	0.10 (0.09 , 0.12)	0.12 (0.10 , 0.13)	0.67 (0.64 , 0.71)	0.65 (0.61 , 0.68)	0.10 (0.09 , 0.12)	0.42 (0.39 , 0.45)
2007	0.11 (0.09 , 0.12)	0.13 (0.11 , 0.14)	0.66 (0.63 , 0.70)	0.59 (0.56 , 0.62)	0.10 (0.09 , 0.12)	0.37 (0.34 , 0.40)
2008	0.11 (0.10 , 0.13)	0.13 (0.12 , 0.15)	0.73 (0.70 , 0.77)	0.65 (0.62 , 0.69)	0.11 (0.09 , 0.12)	0.38 (0.35 , 0.41)
2009	0.11 (0.09 , 0.12)	0.14 (0.13 , 0.16)	0.75 (0.71 , 0.78)	0.67 (0.63 , 0.70)	0.11 (0.10 , 0.13)	0.38 (0.35 , 0.40)
2010	0.11 (0.10 , 0.13)	0.17 (0.15 , 0.19)	0.79 (0.75 , 0.83)	0.70 (0.66 , 0.73)	0.11 (0.09 , 0.12)	0.36 (0.34 , 0.39)
2011	0.13 (0.12 , 0.15)	0.17 (0.15 , 0.19)	0.85 (0.81 , 0.88)	0.70 (0.67 , 0.74)	0.11 (0.10 , 0.13)	0.40 (0.37 , 0.42)
2012	0.14 (0.13 , 0.16)	0.19 (0.18 , 0.21)	0.91 (0.87 , 0.95)	0.71 (0.67 , 0.74)	0.13 (0.11 , 0.14)	0.39 (0.36 , 0.41)
2013	0.14 (0.13 , 0.16)	0.19 (0.18 , 0.21)	1.01 (0.97 , 1.05)	0.73 (0.69 , 0.76)	0.12 (0.10 , 0.13)	0.37 (0.34 , 0.39)
2014	0.17 (0.16 , 0.19)	0.21 (0.19 , 0.23)	1.10 (1.06 , 1.14)	0.81 (0.78 , 0.85)	0.12 (0.11 , 0.13)	0.33 (0.31 , 0.35)
2015	0.17 (0.16 , 0.19)	0.23 (0.21 , 0.24)	1.21 (1.16 , 1.25)	0.87 (0.83 , 0.91)	0.14 (0.13 , 0.16)	0.33 (0.31 , 0.35)

2016	0.17 (0.16 , 0.19)	0.23 (0.21 , 0.25)	1.26 (1.22 , 1.31)	0.85 (0.81 , 0.88)	0.14 (0.13 , 0.16)	0.33 (0.30 , 0.35)
2017	0.17 (0.16 , 0.19)	0.23 (0.21 , 0.25)	1.39 (1.35 , 1.44)	0.93 (0.89 , 0.97)	0.19 (0.17 , 0.21)	0.40 (0.37 , 0.42)
2018	0.18 (0.16 , 0.19)	0.27 (0.25 , 0.29)	1.47 (1.42 , 1.51)	0.94 (0.90 , 0.98)	0.21 (0.19 , 0.23)	0.43 (0.40 , 0.46)
2019	0.18 (0.16 , 0.20)	0.29 (0.26 , 0.31)	1.56 (1.51 , 1.61)	0.98 (0.94 , 1.02)	0.19 (0.18 , 0.21)	0.46 (0.43 , 0.49)
2020	0.28 (0.26 , 0.30)	0.35 (0.33 , 0.38)	1.81 (1.76 , 1.86)	1.15 (1.11 , 1.19)	0.24 (0.22 , 0.26)	0.51 (0.48 , 0.54)
2021	0.32 (0.30 , 0.34)	0.41 (0.39 , 0.44)	2.01 (1.96 , 2.07)	1.31 (1.27 , 1.36)	0.26 (0.24 , 0.28)	0.60 (0.57 , 0.64)
2022	0.27 (0.25 , 0.29)	0.39 (0.37 , 0.42)	1.93 (1.88 , 1.99)	1.25 (1.21 , 1.29)	0.27 (0.25 , 0.29)	0.51 (0.48 , 0.54)
2023	0.23 (0.21 , 0.25)	0.40 (0.37 , 0.42)	1.88 (1.83 , 1.93)	1.08 (1.04 , 1.12)	0.24 (0.22 , 0.26)	0.44 (0.41 , 0.46)
2024	0.22 (0.20 , 0.24)	0.35 (0.33 , 0.37)	1.86 (1.81 , 1.91)	1.04 (1.00 , 1.08)	0.28 (0.26 , 0.30)	0.41 (0.38 , 0.43)

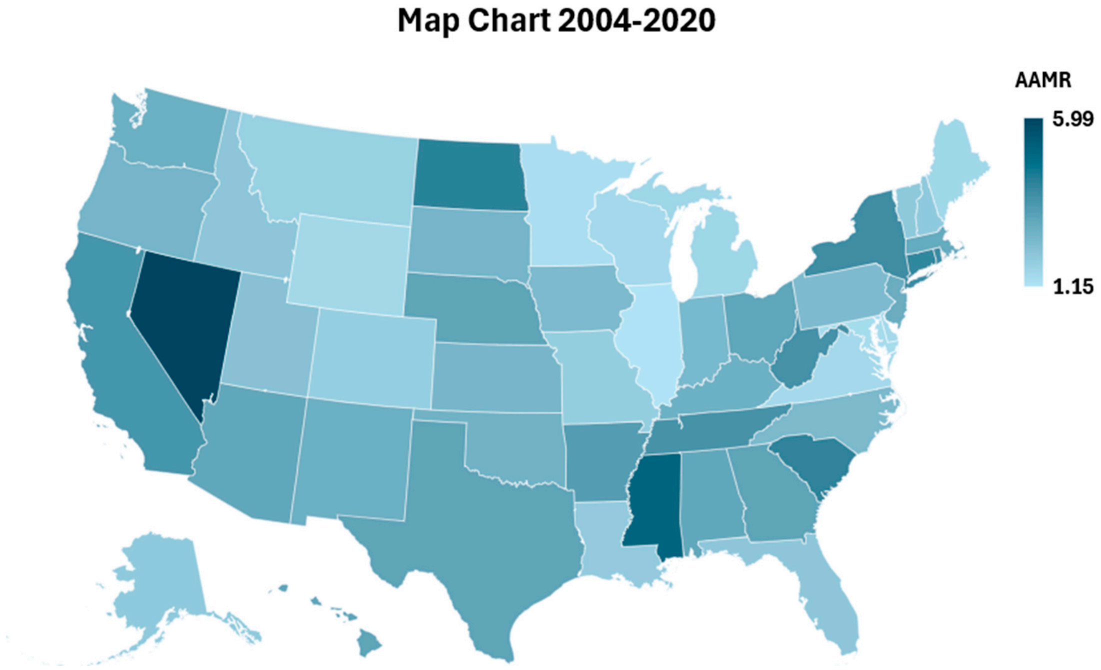

Supplementary Figure S1. State-level age-adjusted mortality rates (AAMRs) for mortality involving cardiac arrest and electrolyte and acid–base disorders among U.S. adults aged ≥25 years, 2004–2020.

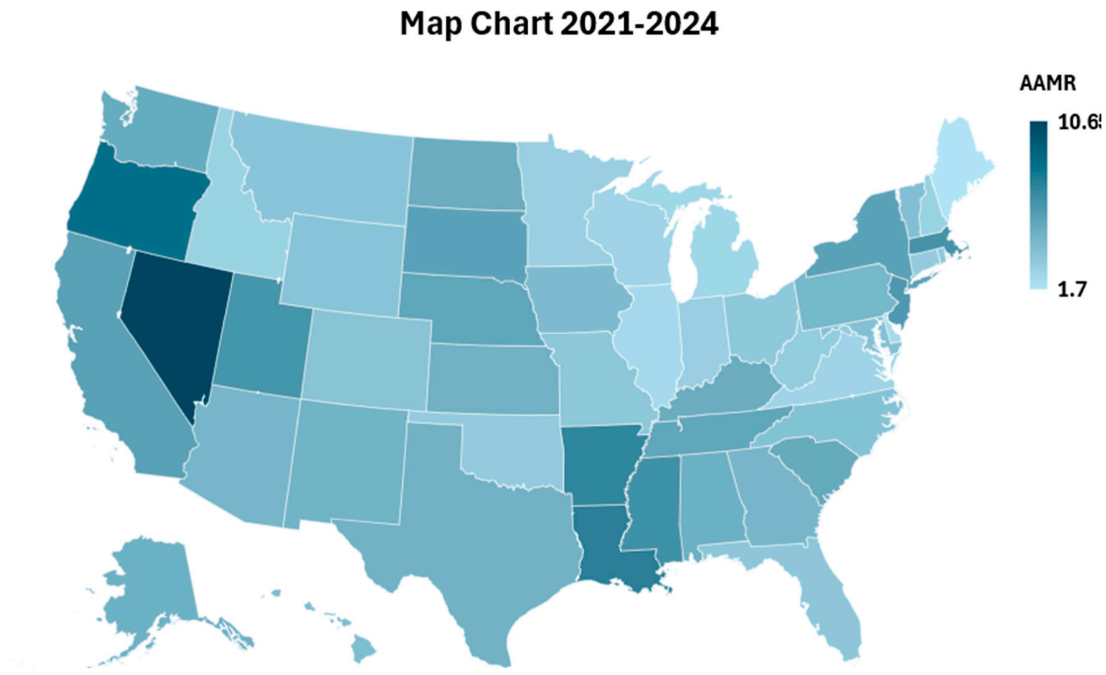

Supplementary Figure S2. State-level age-adjusted mortality rates (AAMRs) for mortality involving cardiac arrest and electrolyte and acid–base disorders among U.S. adults aged ≥25 years, 2021–2024.

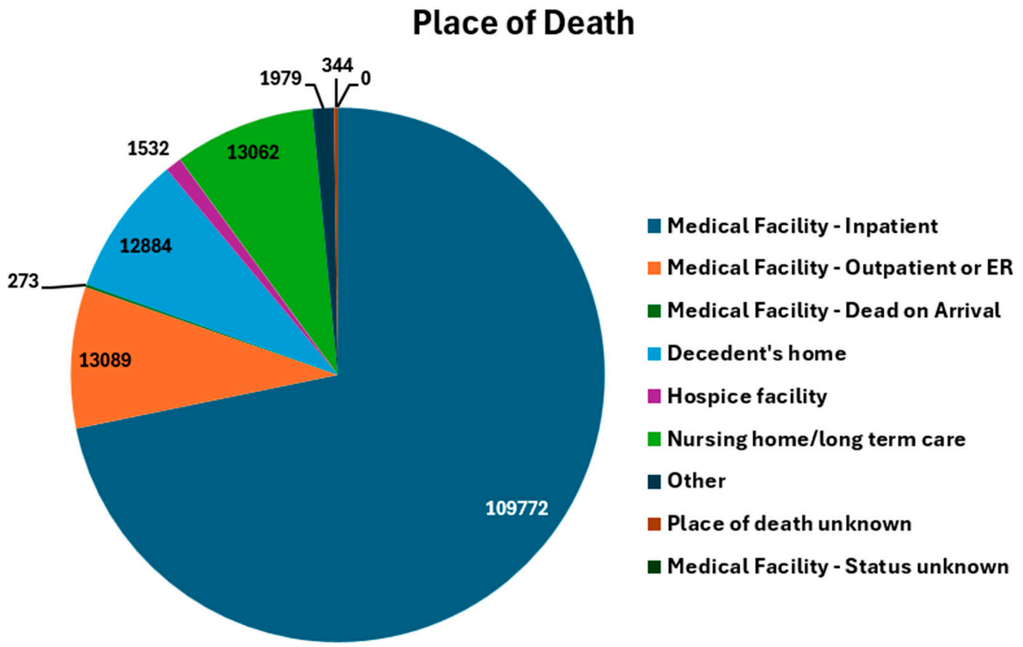

Supplementary Figure S3. Place of death distribution for mortality involving cardiac arrest and electrolyte and acid–base disorders among U.S. adults aged ≥25 years, United States, 2004–2024.

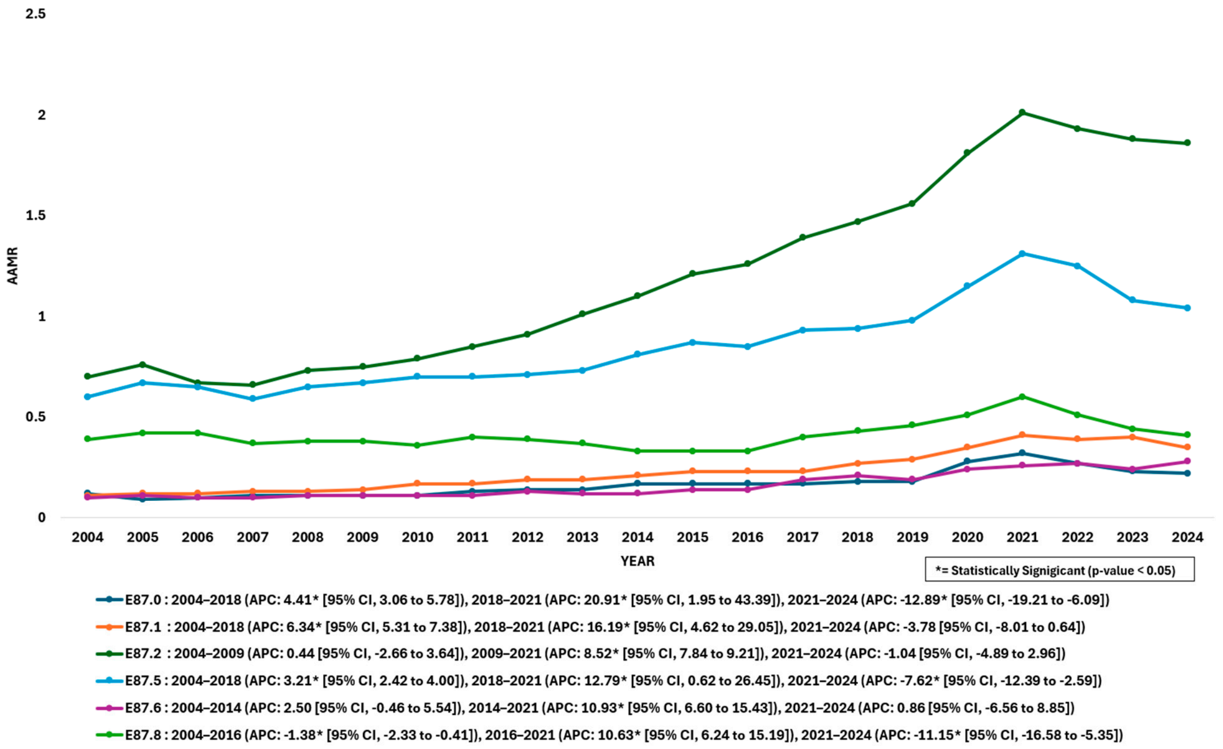

Supplementary Figure S4: Age-adjusted mortality rates according to individual electrolyte and acid–base disorder diagnoses (ICD-10 E87 sub-codes) among deaths involving cardiac arrest in U.S. adults aged ≥25 years, United States, 2004–2024.
